# Chemical and Photochemical-Driven Dissipative Fe^3+^/Fe^2+^-Ion Cross-Linked Carboxymethyl Cellulose Gels Operating Under Aerobic Conditions: Applications for Transient Controlled Release and Mechanical Actuation

**DOI:** 10.1021/jacs.4c00625

**Published:** 2024-03-28

**Authors:** Roberto Baretta, Gilad Davidson-Rozenfeld, Vitaly Gutkin, Marco Frasconi, Itamar Willner

**Affiliations:** † The Institute of Chemistry, The Center for Nanoscience and Nanotechnology, The Hebrew University of Jerusalem, Jerusalem 91904, Israel; ‡ Department of Chemical Sciences, University of Padova, Via Marzolo 1, 35131 Padova, Italy

## Abstract

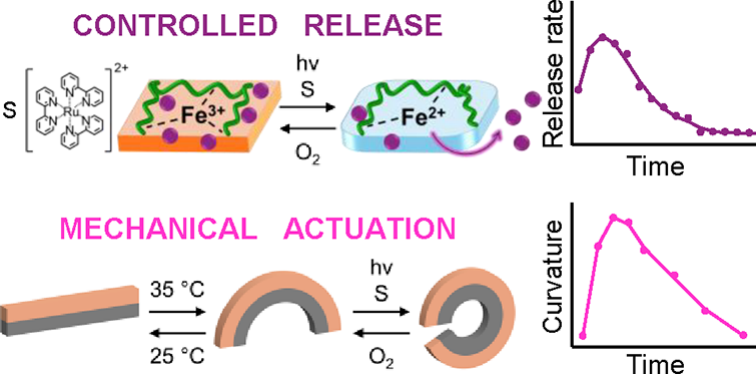

A Fe^3+^-ion cross-linked carboxymethyl cellulose, Fe^3+^-CMC, redox-active gel exhibiting dissipative, transient stiffness properties is introduced. Chemical or photosensitized reduction of the higher-stiffness Fe^3+^-CMC to the lower-stiffness Fe^2+^-CMC gel, accompanied by the aerobic reoxidation of the Fe^2+^-CMC matrix, leads to the dissipative, transient stiffness, functional matrix. The light-induced, temporal, transient release of a load (Texas red dextran) and the light-triggered, transient mechanical bending of a poly-*N*-isopropylacrylamide (p-NIPAM)/Fe^3+^-CMC bilayer construct are introduced, thus demonstrating the potential use of the dissipative Fe^3+^-CMC gel for controlled drug release or soft robotic applications.

## Introduction

Stimuli-responsive hydrogels undergoing signal-triggered reversible stiffness changes attract substantial research efforts on functional materials for diverse applications.^1−5^ These included covalently cross-linked stimuli-responsive hydrogels^6−8^ or hydrogel frameworks cross-linked by stimuli-responsive noncovalent bridges composed of supramolecular complexes^9−14^ or reconfigurable biomolecular bridging units.^1,15^ Different triggers were applied to induce stiffness changes of hydrogels, including physical triggers, such as temperature,^16−18^ light,^19−23^ electrical,^24,25^ magnetic field,^26,27^ or ultrasound irradiation,^28^ and chemical triggers, such as pH,^29−31^ redox agents,^14^ metal ions,^32,33^ donor–acceptor complexes,^34^ and supramolecular ligand–receptor complexes.^35,36^ Also, recent reports demonstrated the triggered self-stiffening and self-softening of hydrogel matrices and their application for shape morphing and mechanoresponse.^37,38^ Diverse applications of stimuli-responsive hydrogels were reported, including their use for sensing,^39,40^ controlled drug delivery and release,^41−44^ shape memory matrices,^45−48^ therapeutic materials for tissue engineering and self-healing,^20,49−51^ and functional materials for actuation and robotic applications.^52−55^ Stimuli-responsive hydrogels are usually composed of polymer scaffolds such as polyacrylamide, polysaccharides such as chitosan, alginate, carboxymethyl cellulose, or polypeptides, to which stimuli-responsive functionalities are tethered as cross-linking units or chemically integrated as part of polymer chains.

An important class of gel materials includes cryogels.^56,57^ Cryogels are prepared under freezing temperatures by analogous chemical procedures to prepare the hydrogels. The cryo-conditions lead to the formation of crystalline solvent domains coated by surrounding concentrated cross-linked polymer scaffolds that, upon defrosting, yield gel matrices with interconnected solute macropores embedded in the polymer framework.^58^ The interconnected macropores comprising the cryogels yield solvent channels in the framework, introducing significant physical properties as compared to the hydrogel, reflected by material compressibility,^59,60^ different mechanical strengths,^61,62^ and most importantly, convection transport of the solute across the channels,^63,64^ as compared to diffusion-controlled transport of the solute in hydrogel matrices. These features lead to substantially faster response times for the stimuli-responsive cryogels as compared to those of analogue hydrogels.^65^

Moreover, an important topic in recent materials science development involves the synthesis of out-of-equilibrium, dissipative, transient operating materials.^66−68^ Particularly, the development of signal-responsive transient operating chemical systems attracts recent research efforts.^69^ For example, the dissipative carbodiimide-fueled synthesis of anhydrides and their hydrolysis,^70^ the hydrolysis of peptides,^71^ or the spatiotemporal assembly and segregation of fibers through the catalytic amination and hydrolysis of peptides^72^ were demonstrated. The concept of dissipative chemical transformations was further applied to tailor the transient dissipative nanostructures and hydrogel matrices. For example, adenosine triphosphate (ATP)-fueled temporal assembly of vesicles and their separation upon hydrolysis of ATP^73^ or the light-induced aggregation of trans-azobenzene monolayer-modified Au nanoparticles upon photochemical isomerization into cis-azobenzene and their separation upon thermal cis to trans isomerization,^74^ were demonstrated. Furthermore, the carbodiimide-fueled cross-linking of a poly(acrylamide-*co*-acrylic acid) solution led to an intermediate hydrogel cross-linked by anhydride bridges that were dissipatively separated to the solution state by hydrolysis of the anhydride bridges.^75^ Also, the supramolecular ferrocene/β-cyclodextrin bridged hydrogel was separated into a solution polymer phase by horseradish peroxidase/H_2_O_2_ catalyzed oxidation of the ferrocene units into the ferrocenium cation state. The competitive ferrocenium cation-mediated oxidation of glucose by glucose oxidase resulted in the dissipative recovery of the hydrogel.^76^ Also, a poly(acrylamide-*co*-acrylic acid) hydrogel cross-linked cooperatively by bis-acrylamide permanent bridges and temporally carbodiimide-generated anhydride bridges demonstrated dissipative transient stiffness changes upon hydrolysis of the anhydride bridges.^77^ The controlled stiffness changes were used to generate the patterned interfaces.

Here, we wish to report on the development of a Fe^3+^/Fe^2+^-cross-linked carboxymethyl cellulose (CMC) redox-reactive dissipative cryogel matrix. We characterize the transient stiffness properties of the cryogel and demonstrate the cyclic transient temporal release of loads from the cryogel and the autonomous mechanical actuation of a soft robotic system. The transient stiffness properties are triggered by ascorbic acid/O_2_ agents. Moreover, the redox-active dissipative cryogel was coupled to a photosensitized electron transfer process reducing the redox sites, allowing the light-induced control of transient stiffness properties of the cryogel and the subsequent light-regulated temporal release of the loads. It should be noted that while redox-triggered switchable stiffness-controlled hydrogels were reported (chemical agents^78^ or electrochemical signals^79^), dissipative redox-active gels are scarce, and particularly, emerging functions of the dissipative matrices are unprecedented.

## Results and Discussion

**Scheme 1:**
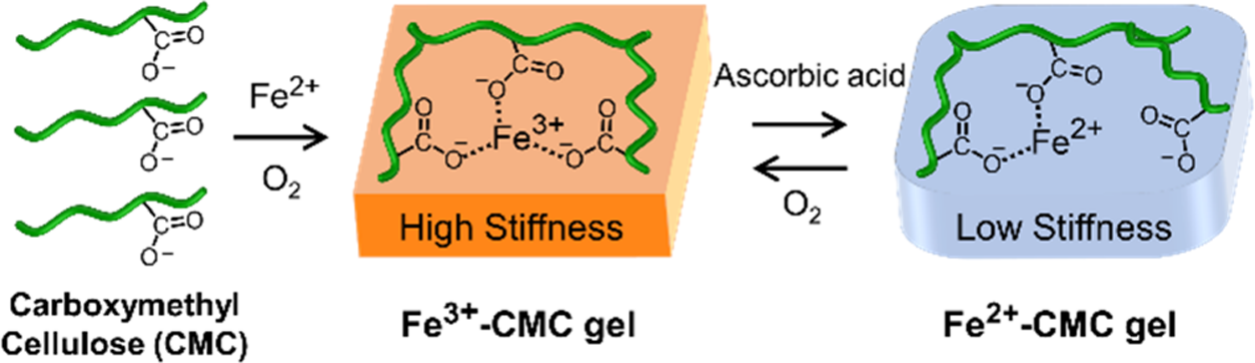
Schematic Preparation of the Fe^3+^-CMC Gel Under Aerobic Conditions and Switchable Stiffness Changes by Reducing and Oxidizing Chemical Agents

Disc-shaped Fe^3+^-cross-linked carboxymethyl cellulose (Fe^3+^-CMC) cryogels were prepared by mixing an aqueous CMC polymer solution with variable concentrations of Fe^2+^–sulfate (FeSO_4_), 20 mM or 40 mM, under nitrogen in a Teflon mold. The resulting solutions were kept under aerobic conditions (−18 °C). To reach a stable cross-linked cryogel framework, the mixture in the mold was thawed and refrozen every 24 h for three cycles (total cross-linking time 4 days). The resulting cryogel extruded from the mold consisted of the Fe^3+^-CMC cross-linked gel (Scheme 1). The formation of the Fe^3+^-CMC gel was confirmed by X-ray photoelectron spectroscopy (XPS) analysis (Figure S1), revealing Fe^3+^ content ≥90%. This procedure to prepare the Fe^3+^-CMC gel was adopted to obtain a disc-shaped configuration for further spectroscopic and rheometric characterizations. The resulting gel prepared in the presence of 40 or 20 mM FeSO_4_ solution revealed, after aerobic oxidation, a 30 mM (higher) and 16 mM (lower) Fe^3+^ gel cross-linking degrees. The properties and functions of the higher cross-linked CMC gel will be presented in the paper, while relevant properties of the lower degree of Fe^3+^ cross-linked gel will be provided in the Supporting Information.

**Figure 1:**
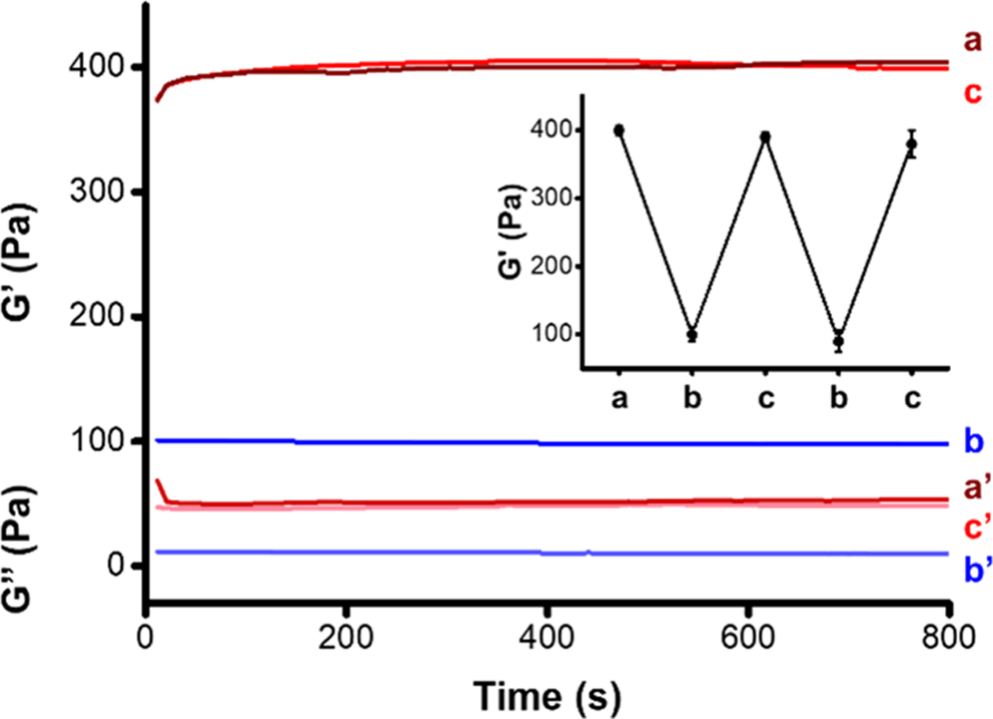
*G*′/*G*″ rheometry parameters corresponding to the 30 mM Fe^3+/2+^-CMC gel: (*a*/*a*′) *G*′/*G*″ values corresponding to the Fe^3+^-CMC gel. (*b*/*b*′) *G*′/*G*″ values of the ascorbic acid-reduced Fe^2+^-CMC gel (using 0.5 mM ascorbic acid). (*c*/*c*′) *G*′/*G*″ values after aerobic reoxidation of the Fe^2+^-CMC to Fe^3+^-CMC (15 h under air). Inset: Cyclic *G*′ changes of the Fe^3+/2+^-CMC upon the reversible reduction/oxidation of the gel.

The Fe^3+^-CMC gels are reduced by ascorbic acid to yield the Fe^2+^-CMC gels. The resulting Fe^2+^-CMC gels, free of ascorbic acid, undergo aerobic oxidation to regenerate Fe^3+^-CMC. The cyclic ascorbic acid reduction/air oxidation processes are reversible, and the redox states of the gels can be confirmed by XPS (Figure S2). The redox-active gels were characterized by rheometry. Figure 1 depicts the stiffness features of the 30 mM Fe^3+^-CMC gel before and after reduction with ascorbic acid. The *G*′/*G*″ value of the Fe^3+^-CMC gel corresponds to *G*′ ≈ 400 Pa and *G*″ ≈ 40 Pa (curve *a*/*a*′). Upon reduction with ascorbic acid, a lower-stiffness Fe^2+^-CMC gel is formed exhibiting *G*′ ≈ 100 Pa, *G*″ ≈ 10 Pa (curve *b*/*b*′). Upon aerobic oxidation, the Fe^3+^-CMC higher-stiffness gel is recovered (curve *c*/*c*′).

The stiffness properties of the gel are reversible (Figure 1, inset). For the rheometric features of the 16 mM Fe^3+^-CMC, see Figure S3. Evidently, the stiffness properties of the Fe^3+^-gels are controlled by the degree of cross-linking of the CMC framework by the bridging Fe^3+^-ions. Moreover, the results depicted in Figure 1 demonstrate that the Fe^3+^-CMC reveals a higher stiffness as compared to the Fe^2+^-CMC. This is consistent with the enhanced three-dentate Fe^3+^ cross-linking of the CMC polymer chains, as compared to the bidentate bridging of the polymer chains in the Fe^2+^-CMC state, as schematically presented in Scheme 1.

**Figure 2:**
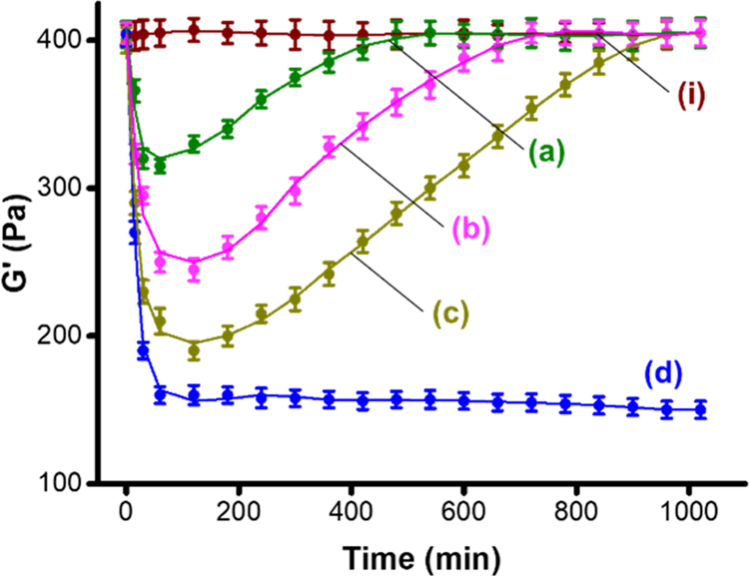
Transient stiffness changes (*G*′) upon the ascorbic acid stimulated reduction of Fe^3+^-CMC to Fe^2+^-CMC and the temporal reoxidation of Fe^2+^-CMC to Fe^3+^-CMC in the presence of (a) 0.033 mM ascorbic acid, (b) 0.066 mM ascorbic acid, (c) 0.1 mM ascorbic acid, and (d) 0.1 mM ascorbic acid under nitrogen. (i) Represents the temporal *G*′-values of the gel in the absence of ascorbic acid under air.

The switchable redox features of the Fe^3+^/Fe^2+^ gel allowed us to apply the redox-responsive gel as a dissipative framework, demonstrating transient stiffness functions as presented in Figure 2. In these experiments, the Fe^3+^-CMC gel was subjected to different concentrations of ascorbic acid, reducing the Fe^3+^-CMC gel to the Fe^2+^-CMC gel (curves (*a*–*c*)). The reduction process is accompanied by a temporal transition of the higher-stiffness Fe^3+^-CMC gel to a lower-stiffness gel composite. As the concentration of ascorbic acid increases, the resulting lower-stiffness values of the composites are more pronounced (for example, in the presence of 0.033 mM ascorbic acid, the gel stiffness drops to *G*′ ≈ 310 Pa, whereas in the presence of 0.1 mM ascorbic acid, the *G*′ value of the gel drops to ≈190 Pa). After reaching the respective ascorbic acid-driven drops in stiffness, the competitive aerobic oxidation of the Fe^2+^-CMC composite proceeds, and the temporal aerobic oxidation of the Fe^2+^-CMC stimulates the transient recovery of the stiffness of the gel framework to the typical *G*′ ≈ 400 Pa value, characterizing the parent Fe^3+^-CMC gel. As the ascorbic acid-driven stiffness changes of the composite are higher, the transient recovery to the parent Fe^3+^-CMC is prolonged. As controls, the Fe^3+^-CMC gel does not show, as expected, any stiffness changes in the absence of ascorbic acid (curve (*i*)). Furthermore, treatment of the gel with ascorbic acid under nitrogen leads to the lower-stiffness Fe^2+^-CMC composite that is not recovered to the parent Fe^3+^-CMC (curve (*d*)), indicating that the transient stiffness properties of the gel, indeed, originate from the aerobic oxidation of the Fe^2+^-CMC framework. For further support of the ascorbic acid/O_2_ Fe^3+^-CMC → Fe^2+^-CMC transition and the dynamic, transient, dissipative transitions Fe^3+^-CMC → Fe^2+^-CMC → Fe^3+^-CMC using absorption spectroscopy, see Figures S4–S6 and the accompanying discussion.

**Figure 3:**
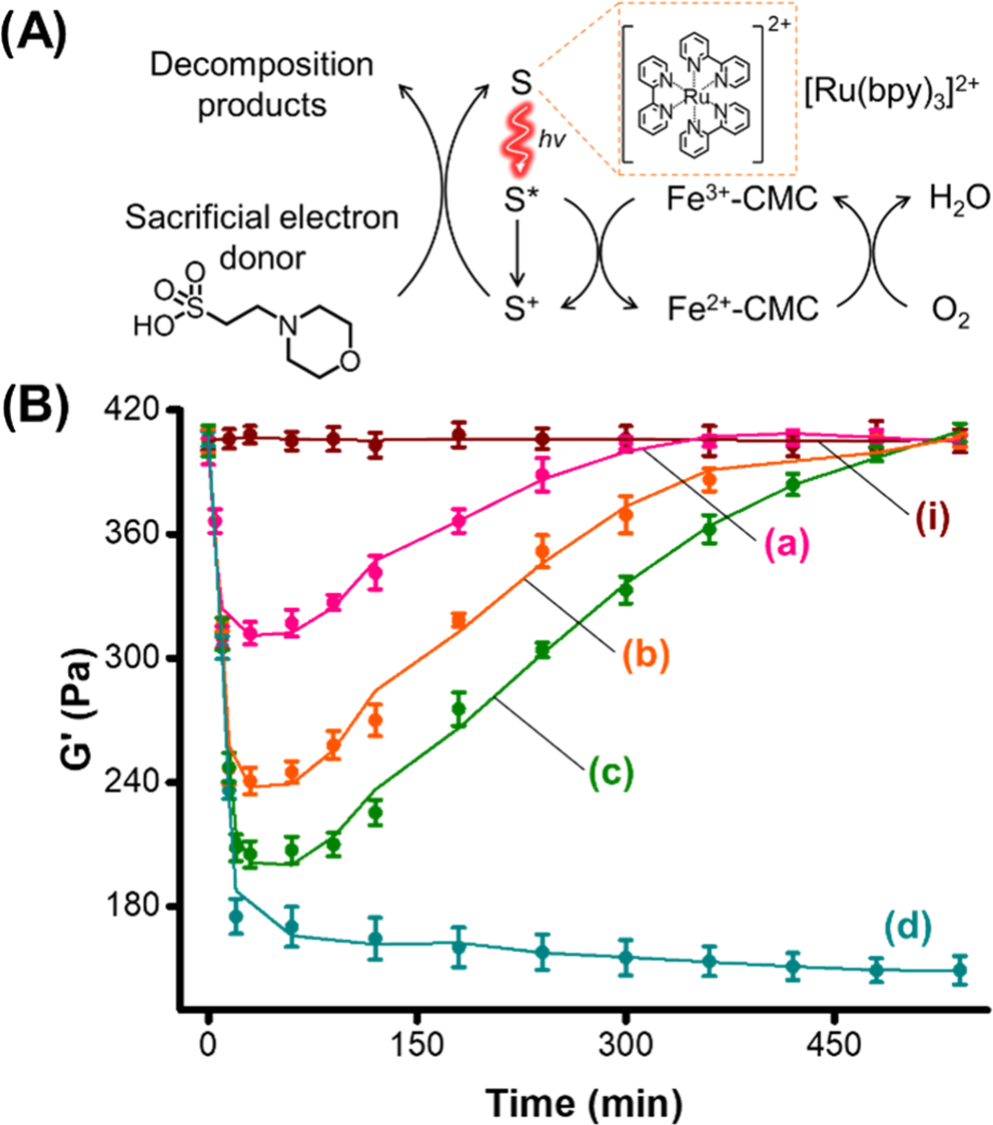
(A) Schematic photosensitized reduction of Fe^3+^-CMC to Fe^2+^-CMC in the presence of the sacrificial electron donor 2-(*N*-morpholino)ethanesulfonic acid and aerobic reoxidation of the gel. (B) Transient stiffness changes (*G*′) upon the photosensitized reduction of Fe^3+^-CMC to Fe^2+^-CMC for different time intervals of LED (λ = 450 nm, 20 mW·cm^–2^) irradiation and subsequent temporal aerobic reoxidation of Fe^2+^-CMC to Fe^3+^-CMC: (a) 10 min irradiation, (b) 15 min, (c) 20 min, and (d) 20 min under nitrogen. (i) Represents the temporal *G*′ values of the gel in the presence of the photosensitizer/electron donor in the dark.

The switchable and transient stiffness properties of the Fe^3+^-CMC cryogel were further triggered by light rather than by chemical reducing agents. Toward this end, we made use of the Ru(II)-tris*-*(bipyridine) [Ru(bpy)_3_]^2+^ complex as a sensitizer^80^ for the [Ru(bpy)_3_]^2+^-photosensitized reduction of the Fe^3+^-CMC to the Fe^2+^-CMC in a 2-(*N*-morpholino)ethanesulfonic acid (MES) buffer solution, acting as a sacrificial electron donor for the reduction of Fe^3+^-CMC to Fe^2+^-CMC, as schematically outlined in Figure 3A. For the photophysical characterizations of [Ru(bpy)_3_]^2+^ in the presence of Fe^3+^-CMC, see Figures S7–S8 and the accompanying discussion. The Fe^3+^-CMC gel in the presence of the MES buffer solution and [Ru(bpy)_3_]^2+^ reveals in the dark higher-stiffness values, *G*′ ≈ 400 Pa, *G*″ ≈ 40 Pa, consistent with the Fe^3+^ state of the gel.

Irradiation of the gel under nitrogen with an LED light source, λ = 450 nm, for 20 min, resulted in a lower-stiffness gel, *G*′ ≈ 150 Pa, *G*″ ≈ 20 Pa, consistent with the photosensitized formation of the Fe^2+^-CMC. Exposure of the Fe^2+^-CMC gel to air resulted after a time interval of 9 h in the recovery of the higher-stiffness gel, consisting of the Fe^3+^-CMC (*G*′ ≈ 400 Pa, *G*″ ≈ 40 Pa). The stiffness changes of the Fe^3+^-CMC gel framework were controlled by the time interval of exposure to the LED irradiation. As the time interval of irradiation was longer, the stiffness of the gel was lower, and it reached a saturation value after ca. 20 min of irradiation (Figure S9). The coupled-light-induced reduction of Fe^3+^-CMC to Fe^2+^-CMC, followed by the aerobic reoxidation of the Fe^2+^-CMC to Fe^3+^-CMC, allowed the development of temporal transient stiffness operating gel. Figure 3B depicts the temporal, transient stiffness curves corresponding to the gel irradiated, λ = 450 nm, for different time intervals (curves (*a*–*c*)). As the time of irradiation is prolonged, a decrease in the stiffness of the gel is further emphasized.

For example, irradiation of the gel for 10 min resulted in a stiffness decrease reflected by lowering the parent stiffness of the Fe^3+^-CMC gel, *G*′ ≈ 400 Pa, to a composite gel, revealing lower stiffness, *G*′ ≈ 300 Pa. In turn, irradiation of the Fe^3+^-CMC gel for 20 min led to a lower-stiffness gel composite, corresponding to *G*′ ≈ 200 Pa. These results are consistent with the enhanced transformation of the Fe^3+^-CMC gel into the Fe^2+^-CMC gel as the irradiation is prolonged. Switching off the light resulted in the temporal, transient stiffness recovery of the Fe^2+^-CMC gels into the parent Fe^3+^-CMC gels. The recovery time intervals are longer as the content of Fe^2+^-CMC is higher, and the recovery time scales are in the range of 4 to 8 h. Control experiments revealed that no stiffness changes in the gels are detected in the dark within this time scale (curve (*i*)). Moreover, irradiation of the gel under nitrogen resulted in a drop in the stiffness of the gel to a constant, saturated value of *G*′ ≈ 180 Pa, indicating that within 20 min of irradiation, the yield of Fe^3+^-CMC conversion into Fe^2+^-CMC is almost completed. Furthermore, the transient recovery of the Fe^2+^-CMC gels under aerobic conditions only confirms the reoxidation of Fe^2+^-CMC to the Fe^3+^-CMC. For the temporal, transient dissipative features of the 16 mM Fe^3+^-CMC gel, see Supporting Information Figure S10 and the accompanying discussion.

**Figure 4:**
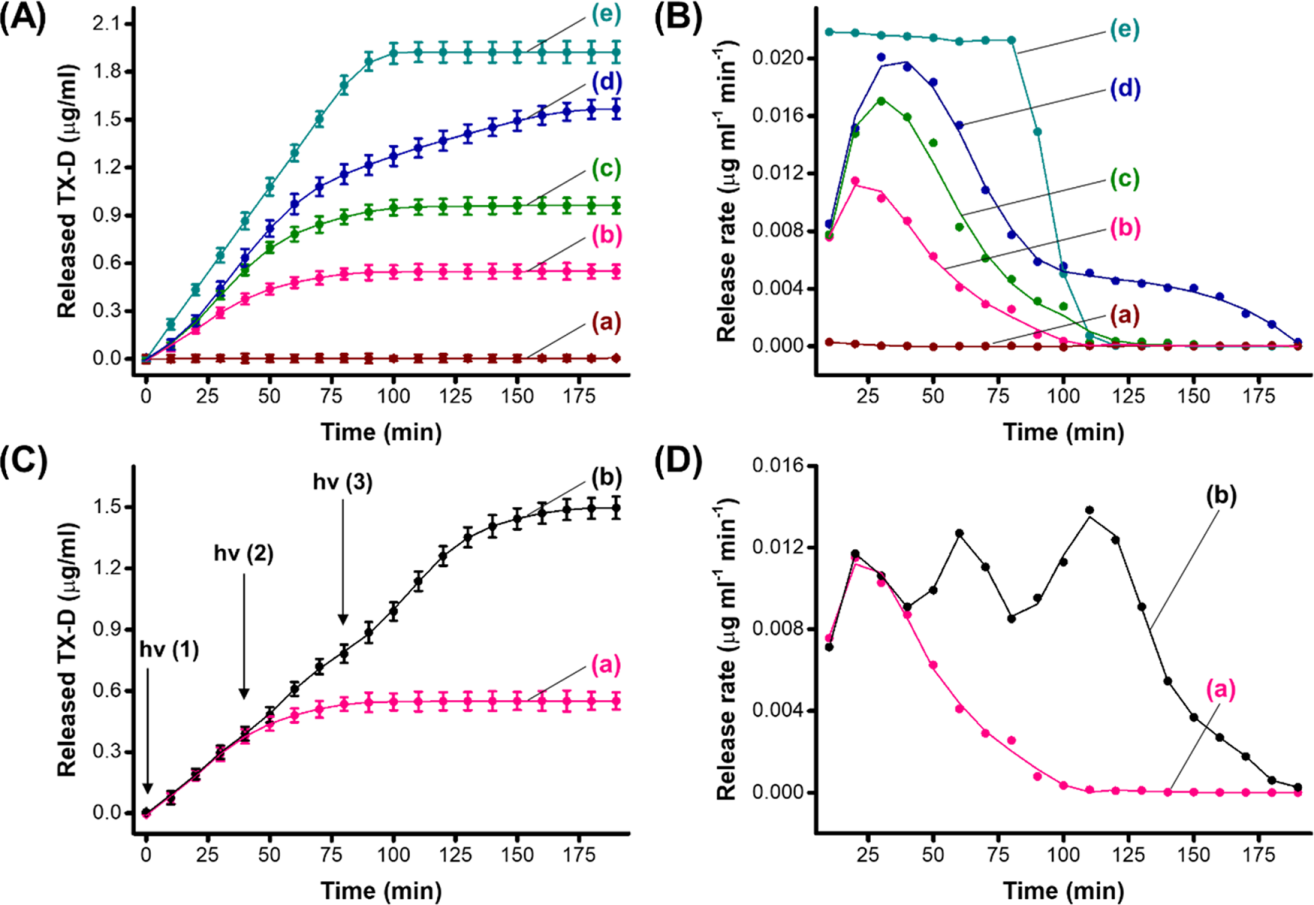
(A) Temporal release of TX-D from the Fe^3+^-CMC gel upon photoirradiation of the gel for different time intervals and allowing the transient aerobic transition of Fe^2+^-CMC to Fe^3+^-CMC that leads to the blockage of the release process. (a) Fe^3+^-CMC in the dark, no irradiation. Illumination of the Fe^3+^-CMC for: (b) 10 min, (c) 20 min, (d) 30 min, and (e) 30 min under N_2_. (B) Rate of release of the TX-D load from the gels, corresponding to the first-order derivative of the curves depicted in panel (A). (C) (a) Temporal release of TX-D from the Fe^3+^-CMC gel upon illumination of the gel for 10 min, followed by the blockage of the release of the load by the dissipative aerobic oxidation of Fe^2+^-CMC to Fe^3+^-CMC. (b) Stepwise switchable temporal release of TX-D upon subjecting the Fe^3+^-CMC gel to ON/OFF illumination cycles. At point (1), the gel is illuminated for 10 min, followed by a time interval of aerobic oxidation of the Fe^2+^-CMC. At point (2), the gel is reilluminated for 10 min and allowed to undergo temporal dark aerobic reoxidation. At point (3), the illumination of the gel is switched on for an additional 10 min, and the resulting gel is allowed to aerobically recover from the Fe^2+^-CMC state to the Fe^3+^-CMC state. (D) (a) Transient, dissipative rates of release of TX-D from the gel upon photoinduced transformation of Fe^3+^-CMC to Fe^2+^-CMC (10 min illumination), followed by transient aerobic recovery of the Fe^2+^-CMC gel to the Fe^3+^-CMC gel in the dark. (b) Switchable cyclic photoinduced transient release rate of TX-D from the gel upon applying cyclic light/aerobic oxidation cycles on the gel. In all experiments, the gel was synthesized by cross-linking CMC with 40 mM Fe^2+^. Light source LED, λ = 450 nm, and 20 mW·cm^–2^.

The temporal dissipative control over the stiffness of the light-triggered Fe^3+^-CMC gel was used to apply the gel as a functional framework for the transient, dose-controlled release of a load. Toward this end, Texas red-modified dextran, TX-D, was loaded in the Fe^3+^-CMC gel matrices, and the light-triggered release of the load from the gel was evaluated. The loading of TX-D in the gel corresponded to 14 μg cm^–3^ of gel. Figure 4A depicts the time-dependent release profiles of the TX-D-loaded Fe^3+^-CMC matrices as a function of the time interval of irradiation. In these experiments, the gel matrices were irradiated for different time intervals to transform the higher-stiffness Fe^3+^-CMC gels into the lower-stiffness composite consisting of Fe^2+^-CMC and yield gels of variable stiffness features that were allowed to follow temporal dissipative aerobic oxidation to the Fe^3+^-CMC, with the vision that the systems could demonstrate temporal, dose-controlled, transient release of the loads. Figure 4A, curve (*a*), demonstrates that no release of TX-D from the higher-stiffness Fe^3+^-CMC proceeds on the time scale of the experiment. Figure 4A, curves (*b*–*d*) show the time-dependent release profiles of TX-D from the 30 mM cross-linked Fe^3+^-CMC gel irradiated, λ = 450 nm, for different time intervals after which the light is switched off and the release profiles of TX-D are monitored along the time interval of the transient, dissipative, aerobic recovery to the Fe^3+^-CMC nonreleasing gel matrices. Evidently, within a time interval of 60–100 min, the load is released, and afterward, the release process reaches saturation, consistent with the recovery of the Fe^3+^-CMC frameworks that prohibited further release of the load. Knowing the loading degree of TX-D in the gel, the temporal release profiles of the load at different time intervals of irradiation of the gel indicate that under irradiation for 20 min, ca. 50% of the load was temporally released from the gel (curve *c*), while illumination of the gel for 30 min, resulted in ca. 85% release of the load (curve *d*).

As the time interval of irradiation is prolonged, the extent of TX-D release increases, reflected by a higher saturation level, where the saturation level indicates the fraction of the load released from the matrix. For comparison, Figure 4A, curve (*e*) depicts the dynamic release profile of TX-D from the gel matrix irradiated for 30 min under nitrogen. A substantial further release of TX-D is observed, which reaches a saturation level after 75 min. Under these conditions, aerobic recovery of the Fe^2+^-CMC matrix is prohibited, and the saturation level of released TX-D can be attributed to the complete release of the load from the framework. Using an appropriate calibration curve, relating the fluorescence intensity of TX-D to its concentration (Figure S11), we estimated that ca. 2.0 μg of TX-D was released from the gel. Accordingly, from the saturation levels observed in curves (*b*–*d*), we estimated that ca. 28, 50, and 85% of the loads integrated in the respective gel matrices were released. Figure 4B depicts the temporal release rates of the TX-D from the Fe^3+^/Fe^2+^-CMC gel matrices irradiated for 10, 20, and 30 min (the release rates correspond to the first-order derivatives of the time-dependent release profiles shown in Figure 4A, curves (*b*–*d*)). Evidently, transient release rate curves are observed, consistent with the dissipative release profile dictated by the stiffness of the gel. That is, the primary light-induced stiffness decrease of the gel upon transition of Fe^3+^-CMC to Fe^2+^-CMC is accompanied by an enhanced triggered release of TX-D reaching a maximum value. The longer the irradiation time of the gel, the degree of stiffness decreases, resulting in a higher content of released TX-D. After reaching a peak release value, the release rates decline and reach a fully blocked release rate. The transient decrease in the release rates of the load is consistent with the competitive aerobic oxidation of Fe^2+^-CMC to Fe^3+^-CMC, a process accompanied by the transient increase in the stiffness of the gel that blocks the release process. Upon complete recovery of the Fe^3+^-CMC process, the release of the load is fully blocked.

Indeed, Figure 4B, curve (*a*) shows that no release of TX-D from the gel in the Fe^3+^-CMC state proceeds. For comparison, the release rates of TX-D from the photoirradiated gel (for 30 min) under nitrogen (Figure 4A, curve (*e*)) are depicted in Figure 4B, curve (*e*). While the release profile of the load revealed a saturation kinetic, the temporal release rates revealed a nondissipative behavior. The irradiation of the gel shows a rapid, constant, high release rate that sharply declines upon reaching saturation and complete release of the load. Furthermore, control experiments illuminating the cryogel in the absence of the [Ru(bpy)_3_]^2+^ photosensitizer did not lead to any release of the TX-D load, implying that the photosensitized reduction of Fe^3+^-CMC to Fe^2+^-CMC, and the accompanying stiffness changes of the gel framework, are mandatory to allow the release of the load.

The transient incomplete release of the load controlled by the dissipative mechanism dictated by the time of irradiation and the competitive aerobic oxidation of the intermediate Fe^2+^-CMC suggested that the release process could also be switched ON and OFF within the transient release of the load. This switchable dissipative control of the release process is depicted in Figure 4C. In this experiment, we irradiated the gel for 10 min and released the TX-D load for a time interval of 40 min. At the time marked with an arrow (2), the gel was irradiated for another 10 min, and the release process of the load was recorded for another time interval of 40 min. After this step of release, at the time marked with an arrow (3), the gel was further irradiated for 10 min, and the release process of TX-D was followed for 110 min. Evidently, each irradiation step is accompanied by an increase in the release of the load, and within the release steps, the release of the load reveals a tendency to saturate, consistent with the dissipative coexistent aerobic oxidation of the gel inhibiting the release process. Figure 4D depicts the temporal change in the rates of the release of the load (TX-D) upon application of the three-step illumination cycles on the gel. Evidently, each step of illumination is accompanied by a transient dissipative release process. For the temporal, transient, dissipative load release from the 16 mM Fe^3+^-CMC gel, see Supporting Information Figure S12 and the accompanying discussion.

**Figure 5:**
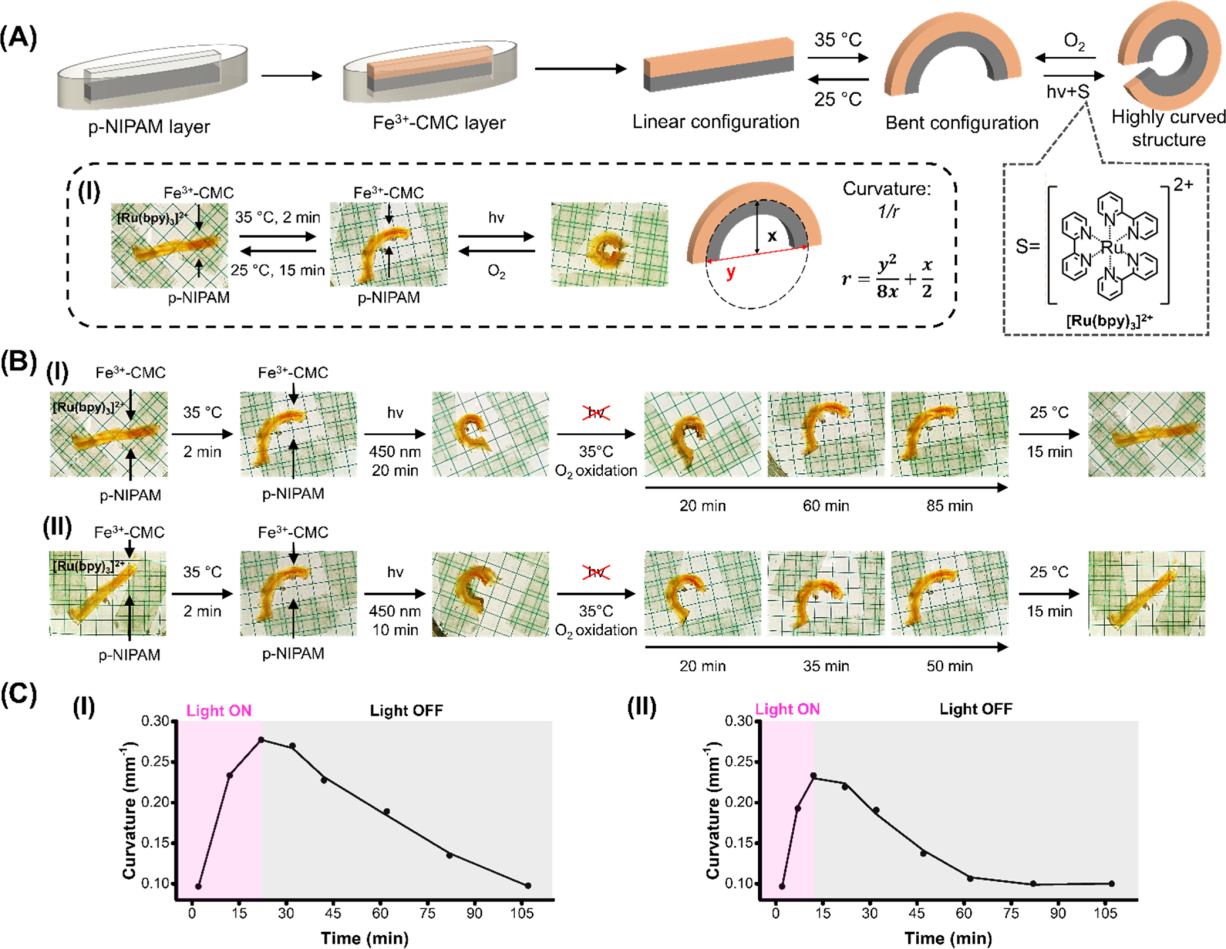
(A) Schematic construction of the p-NIPAM/Fe^3+^-CMC bilayer device and reversible thermoresponsive and photochemical bending of the system. Panel (I): Images of the reversible thermoresponsive and photochemical bending of the bilayer gel device and the schematic evaluation of the bending degree. (B) Time-dependent mechanical bending of the p-NIPAM/Fe^3+^-CMC bilayer device upon thermal and photochemical triggering of the device followed by temporal aerobic oxidation of the p-NIPAM/Fe^3+^-CMC device and recovery of the bilayer linear device. Panel (I): the device is illuminated for 20 min. Panel (II): the device is illuminated for 10 min. (C) Dissipative, transient curvature of the p-NIPAM/Fe^3+^-CMC device upon applying photochemical/aerobic triggers on the device generated by Panel (I): 20 min of illumination. Panel (II): 10 min of illumination. Light source LED, λ = 450 nm, 20 mW·cm^–2^.

The photosensitized control over the stiffness properties of the Fe^3+/2+^-CMC gel and the dissipative aerobic, transient, stiffness-controlled behavior of the gel were then applied to develop an autonomous light-triggered mechanical robotic gel device demonstrating autonomous bending and recovery properties. Toward this end, a bilayer, rod-shape device consisting of the thermoresponsive bis-acrylamide-cross-linked poly-*N*-isopropylacrylamide (p-NIPAM) cryogel layer linked to the Fe^3+^-ion cross-linked CMC cryogel (cross-linked with 30 mM Fe^3+^) layer, was assembled (each layer ca. 1.8 mm thick and 25 mm long) (Figure 5A). The bilayer structure was subjected to a temperature rise from 25 to 35 °C, resulting in the gel-to-solid phase transition of the p-NIPAM cryogel. At 25 °C, the two gel layers exhibit comparable stiffness, *G*′ ≈ 400 Pa, *G*″ ≈ 40 Pa, retaining the bilayer structure in a linear configuration. The thermal gel-to-solid transition resulted in increased stiffness of the p-NIPAM layer, and the resulting difference in the stiffness of the layers led within ca. 2 min to bending of the two layers structure into a bent configuration exhibiting a constant bending curvature corresponding to 0.1 mm^–1^.

The curvature (1/*r*) was evaluated using eq 1, where *r* corresponds to the radius of the bent configuration, *Y* is the distance separating the ends of the curved structure, and *X* is the maximum height of the curved structure.^65,81^
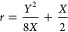
1

The temperature rise from 25 to 35 °C has a small effect on the *G*′ value of the Fe^3+^-CMC gel, *G*′ ≈ 380 Pa. The resulting bent bilayer structure was exposed to 10 or 20 min of irradiation at 35 °C, in the presence of [Ru(bpy)_3_]^2+^ 5 μM in the 20 mM 2-(*N*-morpholino)ethanesulfonic acid (MES) buffer solution to stimulate the photosensitized transition of the higher-stiffness Fe^3+^-CMC to the lower-stiffness Fe^2+^-CMC gel. Afterward, the light source was switched off, and the temporal aerobic oxidation of Fe^2+^-CMC and the recovery of Fe^3+^-CMC at 35 °C were evaluated. The light-induced stiffness changes upon the transition of Fe^3+^-CMC to Fe^2+^-CMC and the subsequent “dark” aerobic transitions of the Fe^2+^-CMC to Fe^3+^-CMC were monitored by following the dynamic curvature changes of the bent “robotic” device. Representative temporal images of bilayer devices illuminated for 20 and 10 min undergoing the light-triggered transition Fe^3+^-CMC to Fe^2+^-CMC and the accompanying aerobic recovery of Fe^2+^-CMC to Fe^3+^-CMC are displayed in Figure 5B, Panels (I, II). Evidently, the linear p-NIPAM/Fe^3+^-CMC bilayer structure undergoes upon heating from 25 to 35 °C rapid bending into a bent configuration, exhibiting a curvature (1/*r*) of ca. 0.1 mm^–1^. That is, an increase of the stiffness of the p-NIPAM layer as compared with the Fe^3+^-CMC layer stiffness led to the observed curvature. The irradiation of the bent structure, λ = 450 nm, led to the [Ru(bpy)_3_]^2+^-photosensitized reduction of the Fe^3+^-CMC layer into the Fe^2+^-CMC layer, exhibiting lower stiffness. That is, the stiffness difference between the p-NIPAM layer and the Fe^3+/2+^-CMC layer was enhanced, resulting in a further bending of the bilayer device. After a time interval of 20 min of irradiation, a circular bent bilayer structure exhibiting a curvature corresponding to 1/*r* ≈ 0.27 mm^–1^ was formed (Figure 5B, Panel (I)). On the other hand, the bilayer gel illuminated for only 10 min (lower content of the Fe^2+^-CMC product) resulted in a lower difference in the stiffness values between the p-NIPAM/Fe^3+/2+^-CMC layers, which led to a lower curvature value of 1/*r* ≈ 0.23 mm^–1^ (Figure 5B, Panel (II)). After the irradiated dynamic bending of the bilayer structures, the systems were allowed to undergo the aerobic reoxidation of the Fe^2+^-CMC constituents to the Fe^3+^-CMC state at 35 °C. As observed, the circular bent bilayer structures undergo a temporal dynamic recovery of the curvatures to the bent bilayer structures, demonstrating the parent curvatures of the systems prior to irradiation. The bilayer device revealing the higher curvature 1/*r* ≈ 0.27 mm^–1^ recovered the original curvature, 1/*r* ≈ 0.1 mm^–1^, after ca. 85 min (Figure 5B, Panel (I)), whereas the circular bent bilayer system generated upon 10 min of irradiation of the device, 1/*r* ≈ 0.23 mm^–1^, recovered to the parent curvature value of 1/*r* ≈ 0.1 mm^–1^ within ca. 50 min (Figure 5B, Panel (II)). Cooling down the resulting bilayer structures to 25 °C restored the linear bilayer configurations of the systems (Figure 5B, Panels (I, II)). Figure 5C depicts the dynamic transient bending curvatures of the bilayer p-NIPAM/Fe^3+^-CMC devices illuminated for 20 and 10 min undergoing photosensitized transition into the p-NIPAM/Fe^3+/2+^-CMC curved states, followed by dynamic, aerobic, dissipative recovery into the p-NIPAM/Fe^3+^-CMC states. Moreover, control experiments supported the suggested mechanism for the photosensitized stiffness changes and the accompanying aerobic dynamic recovery of the stiffness properties of the bilayer devices. In one experiment (Figure S13), the p-NIPAM/Fe^3+^-CMC bilayer structure was subjected to a temperature transition from 25 to 35 °C, resulting in the bent structure, 1/*r* ≈ 0.1 mm^–1^. The resulting structure under air at 35 °C did not show any structural perturbation within 2 h, yet upon cooling to 25 °C, the linear bilayer structure was restored. In a second experiment (Figure S14), the linear bilayer structure at 25 °C was transformed to the bent configuration at 35 °C (1/*r* ≈ 0.1 mm^–1^). The resulting bent structure was irradiated for 20 min, λ = 450 nm, under nitrogen, resulting in a highly curved structure, 1/*r* ≈ 0.30 mm^–1^.

Upon switching off the light and under an anaerobic atmosphere of nitrogen, the gel did not undergo any significant curvature changes for a time interval of ca. 2 h, demonstrating that the reoxidation of the photosensitized Fe^2+^-CMC constituent in the bilayer structure is indeed essential to drive the dynamic recovery of the parent thermally bent system. The highly curved bilayer kept under anaerobic conditions recovered, however, to the parent curved structure upon exposure to air.

Note that we observe differences in the time intervals for the dissipative aerobic recovery of the gels in the mechanical bending experiments and the dissipative load-release and stiffness studies. These differences originate from the different topologies and volumes of the respective gels and the exposed areas of the gels to the aerobic environment. For details, see Supporting Information, Page S20. In fact, in a previous recent report,^65^ the enzyme-catalyzed bending of a bilayer cryogel framework was demonstrated. The novelty of the present system rests, however, on the development of a light-stimulated mechanoresponsive cryogel framework revealing dissipative, transient, and mechanical functions.

## Conclusions

Fe^3+^-ion cross-linked carboxymethyl cellulose (CMC) redox-active gels were introduced as functional, stiffness-responsive, dissipative gel matrices. Chemical or photochemical triggered reduction of the higher stiffness Fe^3+^-CMC gels to the Fe^3+/2+^-CMC lower stiffness gels followed by aerobic reoxidation of the Fe^2+^-CMC gels to the Fe^3+^-state resulted in the transient, dissipative control over the stiffness of the gel matrices. The degree of stiffness changes was controlled by the degree of Fe^3+^-ion cross-linking and by the time interval of the photosensitized transition of the Fe^3+^-CMC to the Fe^2+^-CMC state. The transient, dissipative stiffness functions of the gel matrices were employed for the temporal transient release of loads and for the light-triggered actuation of transient mechanical bending of the bilayer gel constructs.

The importance of the study is reflected by demonstrating new concepts to assemble dissipative gels exhibiting transient stiffness properties. Particularly, the light-induced activation of the material is important for the spatiotemporal activation of the matrices and their potential biomedical application for controlled drug release and soft robotics. Further development of the concept by integrating the photosensitizer into the gel framework and searching for other potential applications of the gels, such as photoresponsive shape memory or light-triggered separation membranes, represents scientific paths to follow.

## Supporting Information Available

Materials and instrumentation; experimental methods; characterizations of gel matrices; absorption spectra; fluorescence spectra; and light-driven experiments (PDF)
