# Supplementary material for: Chemical and Photochemical-Driven Dissipative Fe3+/Fe2+-Ion Cross-Linked Carboxymethyl Cellulose Gels Operating Under Aerobic Conditions: Applications for Transient Controlled Release and Mechanical Actuation
Source: J Am Chem Soc. 2024 Mar 28;146(14):9957–66. doi: 10.1021/jacs.4c00625 (PMC11009950; doi:10.1021/jacs.4c00625)
Supplement: Supplementary file 1 — ja4c00625_si_001.pdf [file ja4c00625_si_001.pdf]

Supporting Information

Chemical and Photochemical Driven Dissipative Fe^{3+}/Fe^{2+}-Ion Crosslinked Carboxymethyl Cellulose Gels Operating Under Aerobic Conditions: Applications for Transient Controlled Release and Mechanical Actuation

Roberto Baretta, †,§ Gilad Davidson-Rozenfeld, † Vitaly Gutkin, † Marco Frasconi,*,§

Itamar Willner *,†

†*The Institute of Chemistry, The Center for Nanoscience and Nanotechnology, The Hebrew University of Jerusalem, Jerusalem 91904, Israel.*

§*Department of Chemical Sciences, University of Padova, Via Marzolo 1, 35131 Padova, Italy.*

*E-mail: itamar.willner@mail.huji.ac.il

*E-mail: marco.frasconi@unipd.it

Table of Contents

1. Experimental Section……………………………………………………………………S2

2. Fe^{3+}-CMC Gel Characterization………………………………………………………S6

3. Redox-Responsive Fe^{3+}-CMC Gel Characterizations……………………………………S7

4. Chemical Driven Transient Dissipative Behavior of Fe^{3+}-CMC Gel……………………S9

5. Photochemical Driven Transient Dissipative Behavior of Fe^{3+}-CMC Gel……………S11

6. Photochemical Driven Transient Release from Fe^{3+}-CMC Matrices…………………S15

7. Photochemical Driven Transient Bending of the Bilayer Device………………………S18

8. Comments on the Time-Scales Corresponding to the Dissipative Recovery of the Different Gel Matrices Under Aerobic Conditions……………………………………………S20

9. References……………………………………………………………………………S21

1. Experimental Section

1.1 Reagents and Materials: All reagents were purchased from commercial suppliers and used without further purification. Sodium carboxymethyl cellulose (CMC, M. W. 250 kDa, degree of substitution (DS) 0.9), iron(II) sulfate heptahydrate ($FeSO_4 \cdot 7H_2O$), L-ascorbic acid, tris(2,2'-bipyridyl)dichloro-ruthenium(II) hexahydrate ($[Ru(bpy)_3]^{2+}$), iron(III) chloride, Texas-Red dextran (TX-D, 70 kDa), 2-(N-morpholino)ethanesulfonic acid (MES), sodium hydroxide, N-isopropylacrylamide (NIPAM), N, N'-methylenebisacrylamide (bis-AAm), N, N, N', N'-tetramethylenediamine (TEMED), and ammonium persulfate (APS, > 98%) were purchased from Sigma Aldrich Co (St. Louis, MO).

1.2 Instrumentation: Ultrapure deionized water from NANOpure Diamond (Barnstead) was employed for all the experiments. Ultraviolet-visible (UV-vis) absorption spectra were recorded with a temperature-controlled UV-2401PC spectrophotometer (Shimadzu, Japan). Time-dependent G'/G'' (Pa) values were measured by a HAAKE MARS III rheometer (Thermo Scientific). Fluorescence spectra were collected with a Cary Eclipse Fluorometer (Varian Inc.), using a quartz cuvette of 1 cm path length. X-ray Photoelectron spectroscopy (XPS) measurements were performed using a Kratos AXIS Supra spectrometer (Kratos Analytical Ltd., Manchester, U.K.) with Al Kα monochromatic radiation X-ray source (1486.6 eV). The XPS spectra were acquired with a takeoff angle of 90° (normal to the analyzer); vacuum condition in the chamber was 2×10^{-9} Torr. High-resolution XPS spectra of Fe 2p were measured with a pass energy of 20 and 0.1 eV step size. The binding energies were calibrated using the C 1s peak energy of 285.0 eV. Data were collected and analyzed by using ESCApe processing program (Kratos Analytical Ltd.) and Casa XPS (Casa Software Ltd.).

1.3 Methods

1.3.1 Gel fabrication: 350 μl of a carboxymethyl cellulose (CMC) 1 % w/w and iron(II) sulfate heptahydrate (Fe^{2+}) solutions (final concentrations of Fe^{2+} 20 mM or 40 mM), previously

degassed with nitrogen for 30 min, were poured in a cylindrical Teflon mold (diameter 12 mm). The mold was stored for 24 h at -18 °C under aerobic conditions, then thawed at room temperature for 45 min. The process of freezing and thawing was then repeated three more times (4 days in total), leading to the formation of an orange gel in the mold. The disc-shaped orange gels were removed from the mold, rinsed with DI water and stored in DI water.

1.3.2 Quantification of Fe^{3+} in the gel: The amount of Fe^{3+} crosslinking the gel was evaluated according to a reported spectrophotometric method.[1] The disc-shaped gels were lyophilized and then transferred into 350 µl of a HCl 2.0 M solution, until complete solubilization of the gel. After a 100-fold dilution in HCl 2.0 M, the UV-vis absorption spectra of the solutions were collected. Fe^{3+} was quantified from a calibration curve obtained with solutions at increasing concentrations of Fe^{3+} in HCl 2.0 M (µM): 135, 169, 210, 281, 420 and 562.

The equation of the calibration curve is: $y = -0.03 + 0.00217\ \mu M^{-1}\ [Fe^{3+}]$; $R^2 = 0.9992$.

1.3.3 Redox-responsive mechanical properties of Fe^{3+}-CMC gel: The mechanical properties of the redox-responsive Fe^{3+}-CMC gels (obtained from Fe^{2+} 40 mM or 20 mM) were evaluated by rheometry, by loading the sample on a temperature controlled parallel plate configuration (20 mm diameter, titanium plates) stage, with fixed temperature of 20 °C, and fixed gap mode (e.g. there is no change in the gap during the measurement), at frequency of 1 Hz and 1 % strain. The gel samples were then transferred into a nitrogen purged 1 ml solution of 0.5 mM ascorbate in 10 mM MES buffer, pH 6.00, and stored under nitrogen. After 1 h, the mechanical properties of the gels were recorded. The gels were exposed to aerobic atmosphere and after 15 h, the mechanical properties of the gels were recorded.

1.3.4 Chemical driven transient, dissipative stiffness properties of Fe^{3+}-CMC gel: The dissipative, transient mechanical properties of the redox-responsive 30 mM Fe^{3+}-CMC gels were evaluated by rheometry, by loading the sample on a temperature controlled parallel plate configuration (20 mm diameter, titanium plates) stage, with fixed temperature of 20 °C, and fixed gap mode at a frequency of 1 Hz and 1 % strain. The gels were immersed into 2 ml of

solution comprising ascorbate at different concentrations, 0.033 mM, 0.066 mM and 0.1 mM, in 10 mM MES buffer, pH 6.00. The mechanical properties were evaluated at different time-intervals by loading the samples on the rheometer.

1.3.5 Photochemical driven transient dissipative stiffness properties of Fe^{3+}-CMC gel: The Fe^{3+}-CMC gels were immersed in 2 ml of a 5 μM $[Ru(bpy)_3]^{2+}$ solution in 10 mM MES buffer, pH 6.00, in the dark. The mechanical properties were evaluated by rheometry, by loading the sample on a temperature controlled parallel plate configuration (20 mm diameter, titanium plates) stage, with fixed temperature of 20 °C, and fixed gap mode at frequency of 1 Hz and 1 % strain. The gel samples immersed in the $[Ru(bpy)_3]^{2+}$ and MES solution were irradiated by a 20 $mW·cm^{-2}$ LED light, λ = 450 nm, for different time-intervals (from 5 to 20 minutes). Immediately after the light was switched off, the gels were moved in the dark, and the mechanical properties were evaluated at different time-intervals by loading the samples on the rheometer.

1.3.6 Loading of Fe^{3+}-CMC gels with TX-D: In order to load TX-D into the gel framework, the fabrication procedure of Fe^{3+}-CMC gels was modified from paragraph "1.3.1 Gel fabrication". A 160 μl solution of CMC, Texas Red-Dextran (TX-D) and $FeSO_4$ (final concentrations: CMC 1 % w/w, TX-D 325 μg/ml and Fe^{2+} 40 mM or 20 mM), previously degassed with nitrogen, was poured in a cylindrical Teflon mold (diameter 8 mm). The mold was stored for 24 h at -18 °C under aerobic conditions, then thawed at room temperature for 45 min. The process of freezing and thawing was then repeated three more times (4 days in total), leading to the formation of purple gels in the mold. The disc-shaped purple gels were removed from the mold and stored in DI water for 48 h prior to use, in order to remove the non-entrapped TX-D.

1.3.7 Photochemical driven transient, dissipative load release: For monitoring the release of TX-D, the disc-shaped Fe^{3+}-CMC gels loaded with TX-D were immersed in a 1 ml solution comprising 5 μM $[Ru(bpy)_3]^{2+}$ in 1 mM MES buffer, pH 6.00, irradiated by a 20 $mW·cm^{-2}$ LED

light, λ = 450 nm, for different time-intervals (from 5 to 30 minutes) and then stored in the dark. Fluorescence spectra of the solution were collected every 10 min using an excitation wavelength of 580 nm and recording the fluorescence emission at 605 nm. TX-D released from the gel was quantified using a calibration curve generated with solutions of TX-D at different concentrations in the presence of 5 μM $[Ru(bpy)_3]^{2+}$ in 1 mM MES buffer, pH 6.00.

1.3.8 Bilayer device fabrication: For the synthesis of the bilayer device, into a rod-shaped Teflon mold (26 mm length, 3 mm width, 5 mm height), 120 μl of a nitrogen purged solution comprising N-isopropylacrylamide (NIPAM), N, N' methylenebisacrylamide (bis-Aam) and ammonium persulfate (APS) were injected and followed by the addition of 40 μl of N, N, N', N'-tetramethylenediamine (TEMED) under nitrogen (final concentrations are 0.5 M NIPAM, 2.5 mM bis-AAm, 17.5 mM APS and 35 mM TEMED). The mold was covered with Parafilm M and the solution degassed for 7 min with nitrogen, to allow the APS initialization. The NIPAM polymerization solution in the mold was then sealed with Parafilm M and stored at -18 °C for a time-interval of two hours. After gelation time, the gel in the mold was thawed at room temperature for 30 minutes in anaerobic environment, followed by three washings of 5 minutes with DI water. Onto the linear p-NIPAM layer obtained, 160 μl of a solution comprising CMC 1% w/w and Fe^{2+} 40 mM, degassed under nitrogen, is added, and the mold was stored at -18 °C for 24 h under aerobic conditions. The mold was allowed to thaw at room temperature for 45 minutes and then three cycles of freezing and thawing were performed (total time 4 days). The linear bilayer p-NIPAM/Fe^{3+}-CMC device was removed from the mold, rinsed with DI water and stored in water.

1.3.9 Bending of the bilayer device: For the bending of the bilayer device, the linear bilayer device was transferred into a 10 ml solution comprising 5 μM $[Ru(bpy)_3]^{2+}$ in 20 mM MES buffer, pH 6.00. Then, the solution was progressively heated and maintained at constant temperature, 35 °C. The bilayer device was allowed to bend until reaching a stable bent configuration. The bent bilayer was then irradiated with 20 $mW\cdot cm^{-2}$ LED light, λ = 450 nm,

for different time-intervals (0, 10 and 20 minutes) and further bending monitored under aerobic atmosphere after switching off the light. After the bilayer device returned to its original bent state at 35°C, the heating of the solution was removed, and the restoration of the bilayer device to the linear configuration was monitored.

2. Fe^{3+}-CMC Gel Characterization

The disc-shaped gels are characterized by X-ray Photoelectron Spectroscopy (XPS). The high-resolution XPS spectra of Fe embedded in the gel frameworks obtained after aerobic exposure of the CMC and Fe^{2+} 40 mM and 20 mM solutions, are depicted in Figure S1A and S1B, respectively. From the XPS spectra, high intensity peaks located at 711 and 724 eV can be observed and can be attributed to Fe^{3+}, while low intensity signals, located at 709 and 723 eV, typical of Fe^{2+}, can be determined.

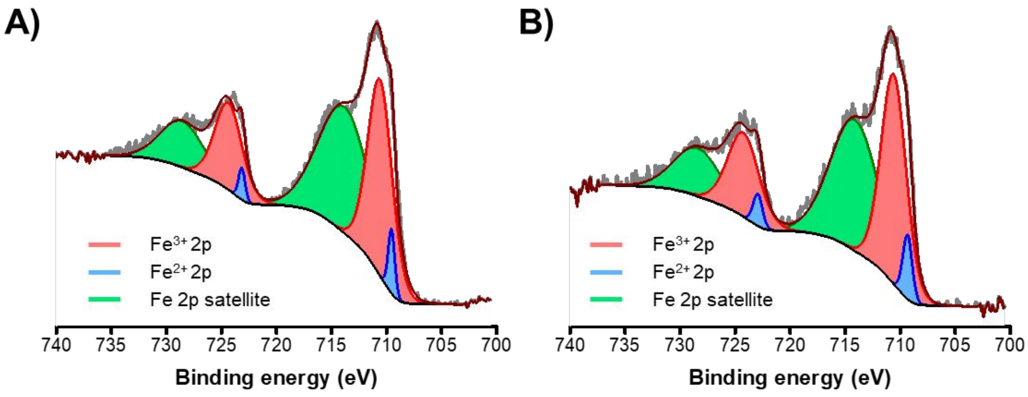

Figure S1. High-resolution Fe 2p XPS spectra (experimental curve in gray, fitted curve in brown) recorded on disc-shaped gels obtained from aerobic oxidation of Fe^{2+} solutions at different concentrations (mM): (A) 40 and (B) 20.

From the ratio between the area under Fe^{2+} and Fe^{3+} peaks, it is possible to determine the percentage of Fe^{3+} crosslinking the gels. For gels obtained from the 40 mM Fe^{2+} solution, the

ratio is 0.080, corresponding to 93 % of Fe^{3+} comprising the gel network, and for gels obtained from the 20 mM Fe^{2+} solution, the ratio is 0.098, corresponding to 91 % of Fe^{3+} comprising the gel network. Therefore, the gels are crosslinked by Fe^{3+}, obtained by aerobic oxidation of Fe^{2+} during the gel fabrication procedure.

3. Redox-Responsive Fe^{3+}-CMC Gel Characterizations

3.1 XPS analysis

The redox-responsive features of the Fe^{3+}-CMC cryogel are demonstrated by XPS. High-resolution XPS spectrum of Fe recorded after the Fe^{3+}-CMC gel is equilibrated in an ascorbic acid 0.5 mM solution under nitrogen is displayed in Figure S2A.

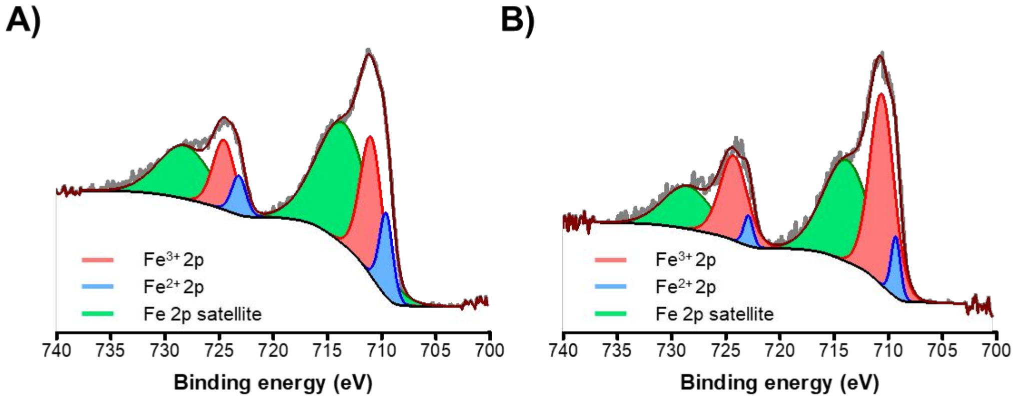

Figure S2. High-resolution Fe 2p XPS spectra (experimental curve in gray, fitted curve in brown) recorded on disc-shaped gels (A) equilibrated in 0.5 mM ascorbic acid solution in 10 mM MES buffer, pH 6.00, under nitrogen and (B) after 15 hours of aerobic oxidation.

The peaks located at 711 and 724 eV, corresponding to Fe^{3+} decrease compared to the initial state (Figure S1), while the peaks at 709 and 723 eV, attributed to Fe^{2+}, increase up to 27 %, indicating that the reduction of Fe^{3+} crosslinker to Fe^{2+} occurred in the gel upon equilibration with an ascorbic acid solution.

Upon exposure of the Fe^{2+}-CMC crosslinked gel to the aerobic atmosphere for 15 hours, the peaks related to Fe^{3+} return to the initial value, before reduction, and the peaks at 709 and 723 eV, attributed to Fe^{2+}, become negligible, (Figure S2B), indicating that aerobic oxidation of Fe^{2+} to Fe^{3+} occurred in the gel network, and restored the parent Fe^{3+}-CMC gel.

3.2 Rheological properties of redox-responsive 16 mM Fe^{3+}-CMC gel

The 16 mM Fe^{3+}-CMC gel is characterized by rheometry (Figure S3), resulting in a G' ≈ 200 Pa and G'' ≈ 20 Pa (curves a/a').

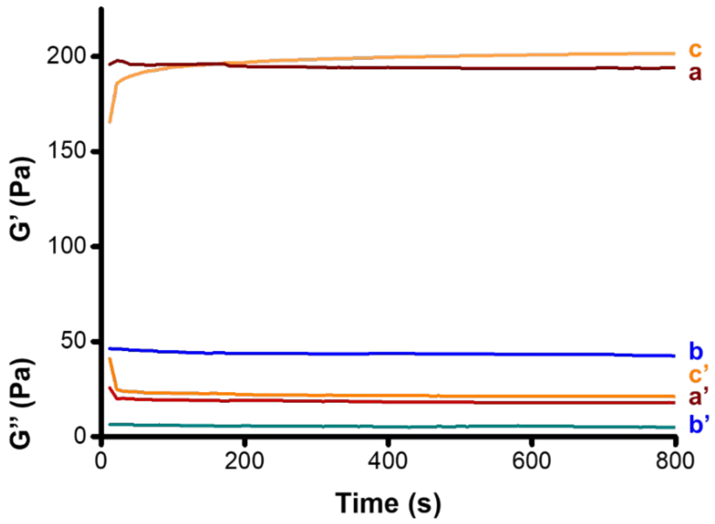

Figure S3. G'/G'' rheometry parameters corresponding to 16 mM Fe^{3+}/Fe^{2+}-CMC gel: (a)/(a') – G'/G'' values of the corresponding to the Fe^{3+}- crosslinked CMC gel. (b)/(b') – G'/G'' values of the ascorbate-reduced Fe^{2+}-CMC gel (using 0.5 mM ascorbate). (c)/(c') – G'/G'' values after aerobic re-oxidation of the Fe^{2+}-CMC to Fe^{3+}-CMC gel (15 h under air).

Upon equilibration in a 0.5 mM ascorbic acid solution in 10 mM MES buffer, pH 6.00, under nitrogen, the resulting composite exhibits a G' ≈ 40 Pa and G'' ≈ 5 Pa (curve b/b'). After exposure of the composite to aerobic atmosphere for 15 hours, the gel displays G' ≈ 200 Pa and

G'' ≈ 20 Pa (curve c/c'), typical of the parent Fe^{3+}-CMC gel. Therefore, the stiffness properties of the redox-responsive 16 mM Fe^{3+}-CMC gel are reversible.

4. Chemical Driven Transient Dissipative Behavior of Fe^{3+}-CMC Gel

The dynamic, transient, dissipative transitions Fe^{3+}-CMC → Fe^{2+}-CMC → Fe^{3+}-CMC induced by ascorbate and O_2 are monitored by UV-vis absorption spectroscopy. The spectrum of the 30 mM Fe^{3+}-CMC gel displays a high intensity absorption ($\lambda < 400$ nm) and a peak located at $\lambda =$ 430 nm (Figure S4), similar to the spectrum of a solution of Fe^{3+} and CMC (Inset, pink curve), indicating that Fe^{3+} is embedded in the gel framework and coordinated by lateral carboxylic groups of CMC chains.

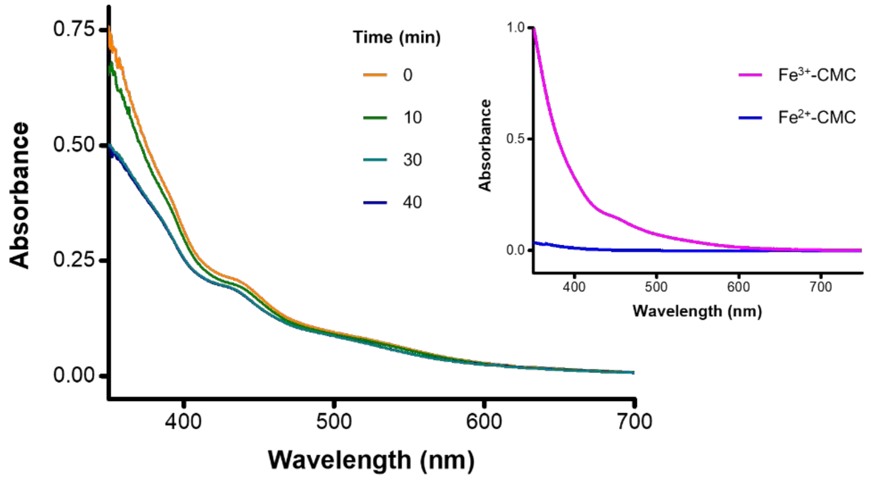

Figure S4. UV-vis absorption spectra collected on 30 mM Fe^{3+}-CMC gel upon addition of 0.066 mM ascorbate in 10 mM MES buffer, pH 6.00, at different time-intervals (min): 0, 10, 30, and 40. Inset: UV-vis absorption spectra of a solution of Fe^{3+} and CMC (pink) and Fe^{2+} and CMC (blue).

Upon addition of ascorbate, the absorbance values of the gel progressively decrease. Indeed, ascorbate promotes the reduction of Fe^{3+}-CMC complex into Fe^{2+}-CMC, which does not display any significant absorption peak in the region $\lambda < 430$ nm, as observed from the spectrum

of a CMC solution in the presence of Fe^{2+} ions (Inset, blue curve). Therefore, the absorbance of the Fe^{3+}-CMC gel progressively decreases after the addition of ascorbate due to the reduction of Fe^{3+}-CMC into Fe^{2+}-CMC, until constant absorption is obtained after 40 min from the ascorbate addition.

After reaching an absorption minimum in about 40 min , the absorbance values collected for the Fe^{2+}-CMC gel progressively increase, as shown in Figure S5.

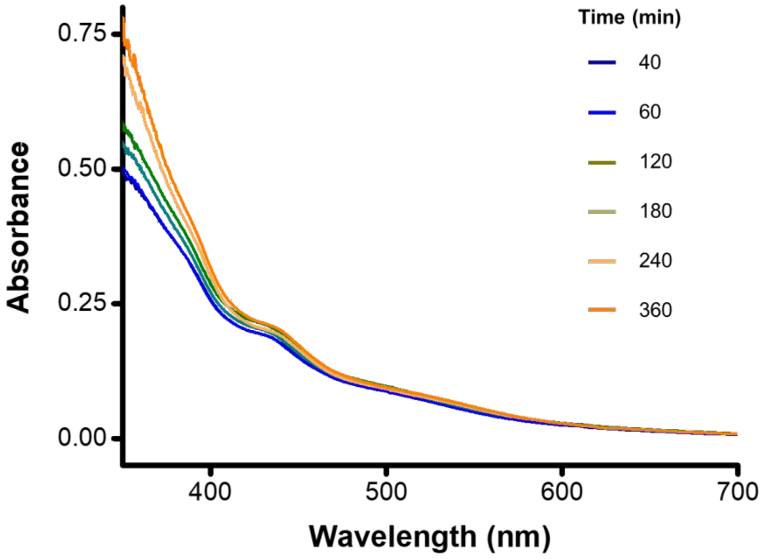

Figure S5. UV-vis absorption spectra collected on 30 mM Fe^{3+}-CMC gel upon addition of 0.066 mM ascorbate in 10 mM MES buffer, pH 6.00, at different time-intervals (min): 40, 60, 120, 180, 240, and 360.

The increase in the absorbance of the gel is provided by the aerobic oxidation of the colorless Fe^{2+}-CMC complex into the orange Fe^{3+}-CMC. The absorbance values recorded for the gel increase, and after 360 min upon the addition of ascorbate, the spectrum of the gel displays absorbance values similar to the spectrum collected on the parent Fe^{3+}-CMC gel. Therefore, O_2 provides a quantitative oxidation of Fe^{2+} to Fe^{3+} in the CMC network during this time-interval, restoring the parent Fe^{3+}-CMC gel, and no leakage of Fe ions occurred during the ascorbate driven dissipative process.

The changes in absorbance at 360 nm for the Fe^{3+}-CMC gel upon reduction by ascorbate and concomitant re-oxidation by O_2 are reported in Figure S6. After the fast initial drop in the absorbance, induced by the ascorbate driven reduction of Fe^{3+}-CMC into the colorless Fe^{2+}-CMC, the aerobic oxidation of Fe^{2+} to Fe^{3+} induces an increase in the absorbance, until the value of the parent Fe^{3+}-CMC gel is restored.

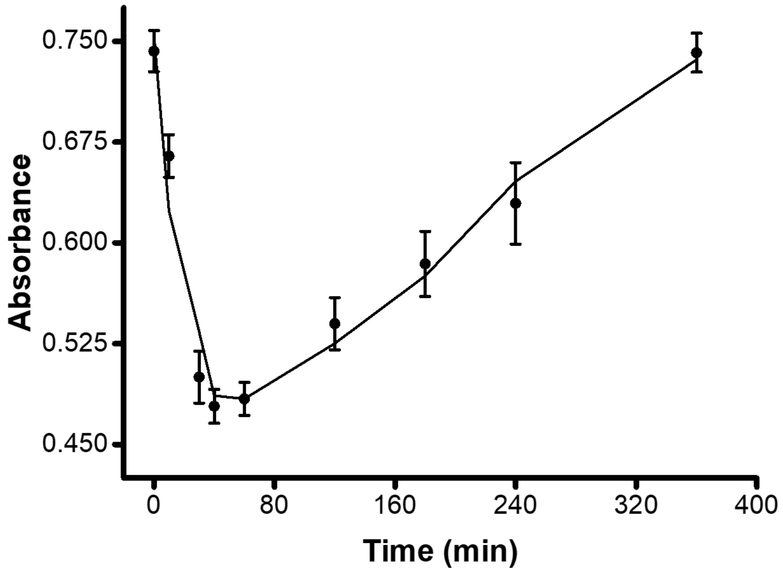

Figure S6. Time dependence absorbance changes (λ = 360 nm) in the absorption spectra collected on the 30 mM Fe^{3+}-CMC gel upon addition of 0.066 mM ascorbate solution in 10 mM MES buffer, pH 6.00.

5. Photochemical Driven Transient Dissipative Behavior of Fe^{3+}-CMC Gel

5.1 Spectroscopic characterizations

The fluorescence spectrum of a 5 μM $[Ru(bpy)_3]^{2+}$ in a 0.1% w/w CMC solution (λ_{exc} = 450 nm) displays a maximum emission (I_0) located at 609 nm. By addition of 62.5 μM Fe^{3+} in the solution, the fluorescence intensity (I) of $[Ru(bpy)_3]^{2+}$ decreases, and by further additions of Fe^{3+} in the CMC and $[Ru(bpy)_3]^{2+}$ solution, a larger decrease in the fluorescence intensity (I)

can be recorded, indicating that Fe^{3+} acts as a quencher for photosensitized $[Ru(bpy)_3]^{2+}$ (Figure S7A). The fluorescence of photosensitized $[Ru(bpy)_3]^{2+}$ is quenched due to the Ru-mediated electron transfer to Fe^{3+}-CMC, which is therefore reduced to Fe^{2+}-CMC.

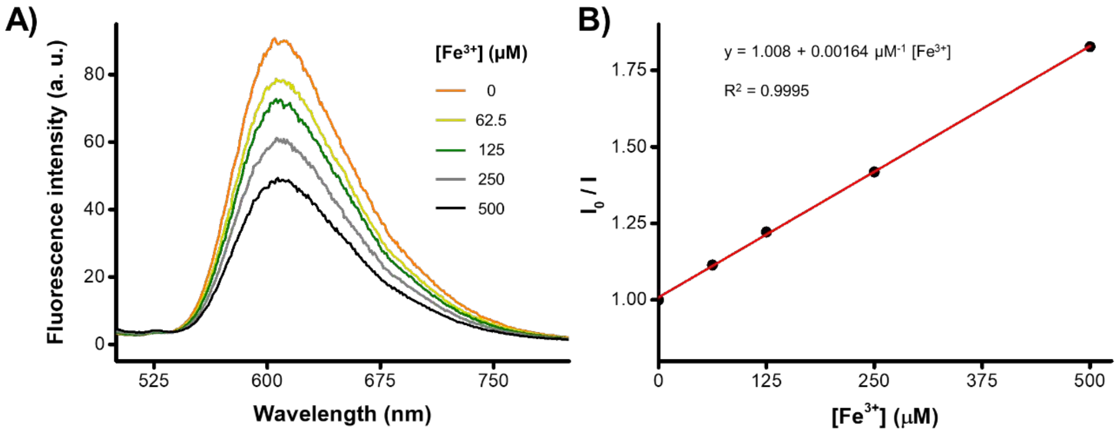

Figure S7. (A) Fluorescence spectra of a 5 μM $[Ru(bpy)_3]^{2+}$ and 0.1 % w/w CMC solution in the presence of Fe^{3+} at different concentrations (μM): 0, 62.5, 125, 250 and 500. (B) Stern-Volmer plot for the quenching of the $[Ru(bpy)_3]^{2+}$ emission in the presence of Fe^{3+}-CMC.

The second-order rate constant for the electron transfer process (k_q) can be calculated by the ratio of the Stern-Volmer constant, obtained from the slope of the fluorescence intensity ratio I_0/I as a function of Fe^{3+} concentration (Figure S7B), and the lifetime of photosensitized $[Ru(bpy)_3]^{2+}$ equal to 0.60 μs,[2] providing $k_q = 2.73 \times 10^9\ M^{-1} \cdot s^{-1}$.

The quenching of the fluorescence of $[Ru(bpy)_3]^{2+}$ upon addition of Fe^{3+} cannot be observed in the presence of 10 mM 2-(N-morpholino)ethanesulfonic acid (MES) buffer (Figure S8), because the tertiary amino group of MES acts as a sacrificial electron donor to restore the $[Ru(bpy)_3]^{2+}$. Indeed, after the photosensitized $[Ru(bpy)_3]^{2+}$ transfers an electron to Fe^{3+}-CMC, it is oxidized to $[Ru(bpy)_3]^{3+}$, which displays no fluorescence. The amino group of MES acts as a sacrificial electron donor to restore the $[Ru(bpy)_3]^{2+}$, which displays fluorescence emission upon irradiation.

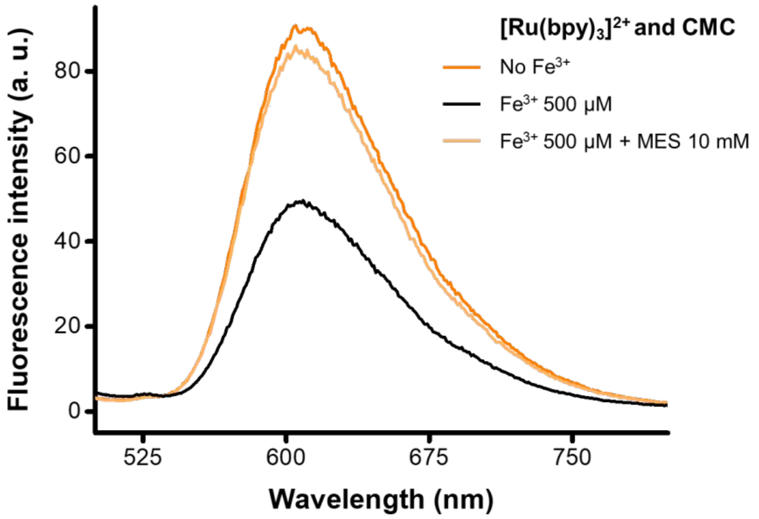

Figure S8. Fluorescence spectra of 5 µM $[Ru(bpy)_3]^{2+}$ and 0.1 % w/w CMC solution in the absence (orange) and in the presence of 500 µM Fe^{3+} (black) and upon addition of 10 mM MES buffer (light orange).

5.2 Optimization of the irradiation time

The optimal irradiation time-intervals for the dissipative transient stiffness changes are evaluated on the Fe^{3+}-CMC gel crosslinked by 30 mM Fe^{3+}. Without irradiation, the gel in a solution of 5 µM $[Ru(bpy)_3]^{2+}$ and 10 mM MES buffer, pH 6.00, as sacrificial electron donor, displays G' ≈ 400 Pa, typical of its fully oxidized state (Figure S9). Upon irradiation (λ = 450 nm, 20 mW·cm^{-2}), G' values of the gel progressively decrease, and the difference in stiffness is enhanced for prolonged irradiation time-intervals. Upon 20 minutes of irradiation, a value of G' ≈ 200 Pa is recorded, and the G' value does not significantly decrease for longer irradiation time (25 minutes and 30 minutes). Therefore, 20 minutes is chosen as the maximum irradiation time for evaluating the transient stiffness properties of the Fe^{3+}-CMC gel.

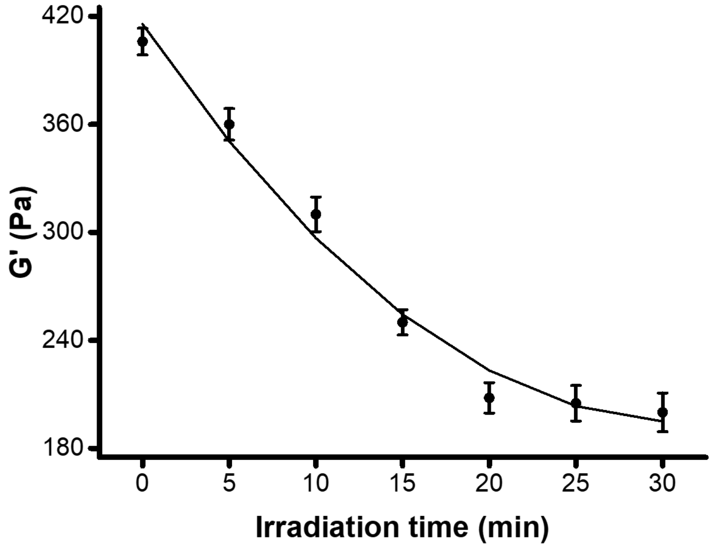

Figure S9. Stiffness changes (G') of the $Fe^{3+/2+}$-CMC gel upon irradiation, (λ = 450 nm, 20 mW·cm^{-2}), in a 5 μM $[Ru(bpy)_3]^{2+}$ and 10 mM MES buffer, pH 6.00, at different time-intervals (min): 0, 5, 10, 15, 20, 25 and 30.

5.3 Transient stiffness properties of the 16 mM Fe^{3+}-CMC gel

The Fe^{3+}-CMC gel, crosslinked by 16 mM Fe^{3+}, in the presence of the MES buffer solution and $[Ru(bpy)_3]^{2+}$ reveals in the dark stiffness values of G' ≈ 200 Pa and G'' ≈ 20 Pa, consistent with the Fe^{3+}-state in the gel (Figure S10, curve (i)). The stiffness changes of the Fe^{3+}-CMC gel framework are induced by the time-interval of exposure to the LED irradiation, for 5, 10 and 15 minutes (curves (a)-(c)). As the time of irradiation is prolonged, the decrease in the stiffness of the gel is further emphasized. Irradiation of the gel for 5 minutes, results in a stiffness decrease to G' ≈ 170 Pa. In turn, irradiation of the Fe^{3+}-CMC gel for 15 minutes leads to a lower stiffness gel composite, corresponding to G' ≈ 110 Pa. These results are consistent with the enhanced transformation of the Fe^{3+}-CMC gel into the Fe^{2+}-CMC gel as the irradiation is prolonged. Switching off the light results in the temporal, transient stiffness recovery of the Fe^{2+}-CMC gels into the parent Fe^{3+}-CMC gels. The recovery time-intervals are longer, as the Fe^{3+}-CMC gel is irradiated for longer times, and the recovery time scales are in the range of 6

to 9 hours. Moreover, irradiation of the gel under nitrogen results in a drop in the stiffness of the gel to a constant value of G' ≈ 90 Pa.

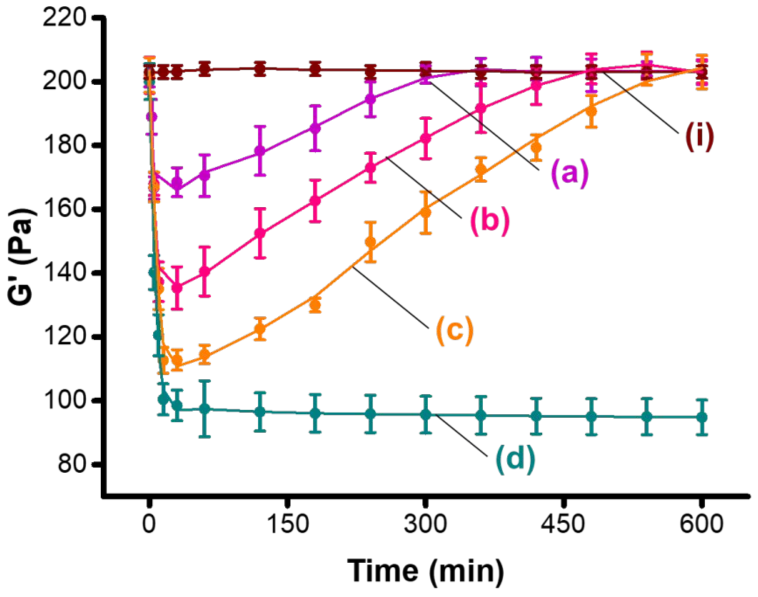

Figure S10. Transient stiffness changes (G') upon the photosensitized reduction of Fe^{3+}-CMC gel to Fe^{2+}-CMC for different time-intervals of LED (λ = 450 nm, 20 mW·cm^{-2}) irradiation and subsequent temporal aerobic re-oxidation of Fe^{2+}-CMC to Fe^{3+}-CMC: (a) 5 minutes of irradiation, (b) 10 minutes, (c) 15 minutes and (d) 15 minutes under nitrogen. (i) Temporal G' values of the gel in the presence of 5 µM $[Ru(bpy)_3]^{2+}$ and 10 mM MES buffer, pH 6.00, in the dark.

6. Photochemical Driven Transient Release from Fe^{3+}-CMC Matrices

The amount of the released Texas-Red dextran (TX-D) was determined by a calibration curve (Figure S11) in which the fluorescence intensities of solutions of TX-D were correlated to the concentration of TX-D, expressed in µg/ml, in a solution comprising 5 µM $[Ru(bpy)_3]^{2+}$ and 1 mM MES buffer, pH 6.00.

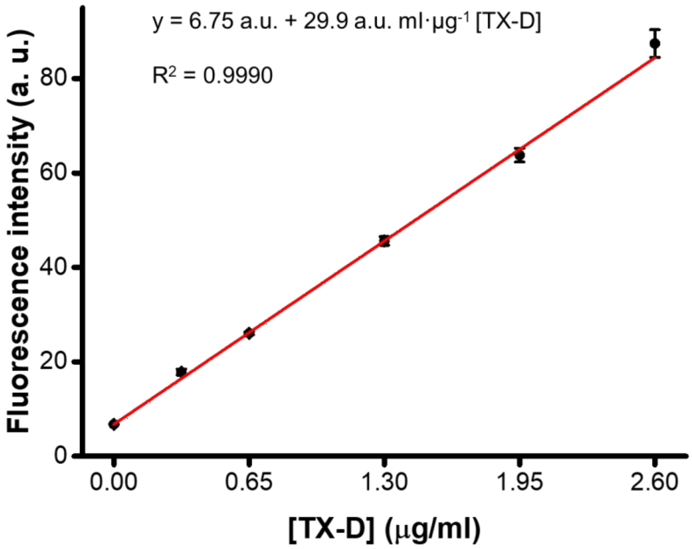

Figure S11. Calibration curve obtained from the fluorescence intensities of 5 μM $[Ru(bpy)_3]^{2+}$ and 1 mM MES buffer, pH 6.00, solutions in the presence of increasing amounts of TX-D (μg/ml): 0, 0.33, 0.65, 1.30, 1.95 and 2.60.

6.1 Transient TX-D release from the 16 mM Fe^{3+}-CMC gel

The time-dependent release profiles of the TX-D-loaded on 16 mM Fe^{3+}-CMC matrices as a function of the time-interval of irradiation are depicted in Figure S12A. Curve (a), demonstrates that no release of TX-D from the higher stiffness 16 mM Fe^{3+}-CMC proceeds in the dark on the time scale of the experiment. Curves (b) and (d) show the time dependent release profiles of TX-D from the Fe^{3+}-CMC gel irradiated for 5 minutes and 10 minutes respectively, after which the light is switched off and the release profiles of TX-D are monitored. As the time-interval of irradiation is prolonged, the extent of TX-D release increases. For comparison, curve (e) depicts the dynamic release profile of TX-D from the gel matrix irradiated for 10 minutes under nitrogen. A substantial further release of TX-D is observed, which reaches a saturation level after 100 minutes. Under these conditions, the aerobic recovery of the Fe^{2+}-CMC matrix is prohibited, and the saturation level of released TX-D can be attributed to the complete release of the load from the framework, which is estimated up to ca. 2.1 μg/ml. Accordingly, from the

saturation levels observed in the curves (b) and (d), we estimate that ca. 19 % and 66 % of the loads integrated in the respective gel matrices are released. The release process can also be switched ON and OFF within the transient release of the load, as depicted by curve (c). We irradiate the gel for 5 minutes and release the TX-D load for a time-interval of 40 minutes. The gel was then irradiated for another 5 minutes, and the release process of the load is followed for 135 minutes. Evidently, each irradiation step is accompanied by an increase in the release of the load, and within the release steps, the release of the load reveals a tendency to saturate, consistent with the dissipative coexistent aerobic oxidation of the gel inhibiting the release process.

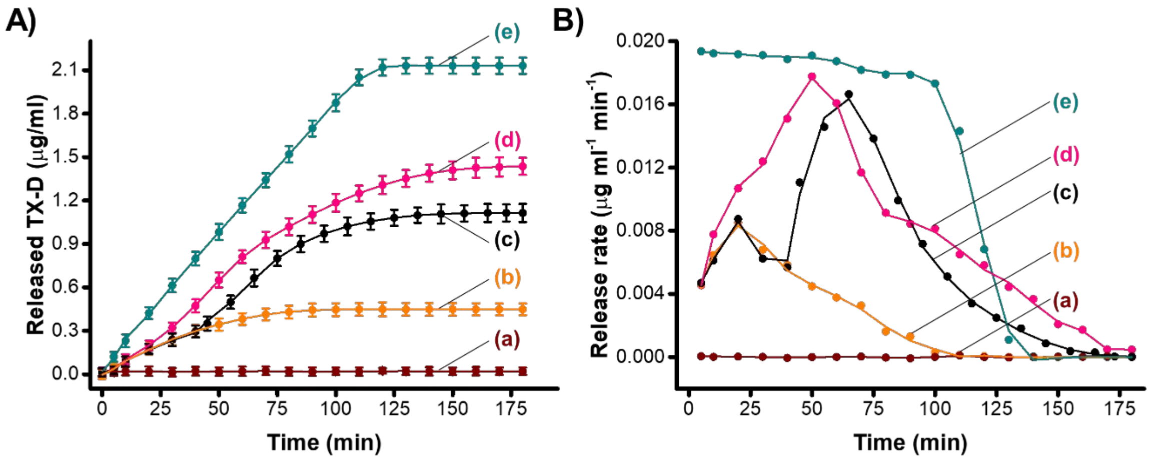

Figure S12. (A) Temporal release of TX-D from the 16 mM Fe^{3+}-CMC gel upon photoirradiation of the gel for different time intervals and allowing transient aerobic transition of Fe^{2+}-CMC to Fe^{3+}-CMC that leads to the blockage of the release process. (a) Fe^{3+}-CMC in the dark. Illumination of the Fe^{3+}-CMC for: (b) 5 minutes, (c) 5 minutes of switchable ON/OFF illumination cycles, (d) 10 minutes, (e) 10 minutes under N_2. (B) Rate of release of the TX-D load from the gels corresponding to the first order derivatives of the curves depicted in (A).

Figure S12B depicts the temporal release rates of the TX-D from the Fe^{3+}/Fe^{2+}-CMC gel matrices irradiated for different time intervals (the release rates correspond to the first-order derivatives of the time-dependent release profiles shown in Figure 12A). Evidently, transient

release rate curves are observed, consistent with the dissipative release profile dictated by the stiffness of the gel. The longer the irradiation-time of the gel, the degree of stiffness decreases, resulting in a higher content of released TX-D. After reaching a peak release value, the release rates decline and reach a fully-blocked release rate, upon complete recovery of the Fe^{3+}-CMC. Indeed, curve (a) shows that no release of TX-D from the gel in the Fe^{3+}-CMC state proceeds. For comparison, the release rates of TX-D from the photo-irradiated gel (for 10 minutes) under nitrogen, (curve (e)), reveal a non-dissipative behavior. The irradiation of the gel shows a rapid, constant high release rate that sharply declines upon reaching saturation and complete release of the load.

7. Photochemical Driven Transient Bending of the Bilayer Device

The linear p-NIPAM/Fe^{3+}-CMC bilayer device was immersed in a solution comprising 5 µM $[Ru(bpy)_3]^{2+}$ and 20 mM MES buffer, pH 6.00, and subjected to an increase in temperature up to 35 °C for 2 hours, as depicted in Figure S13A.

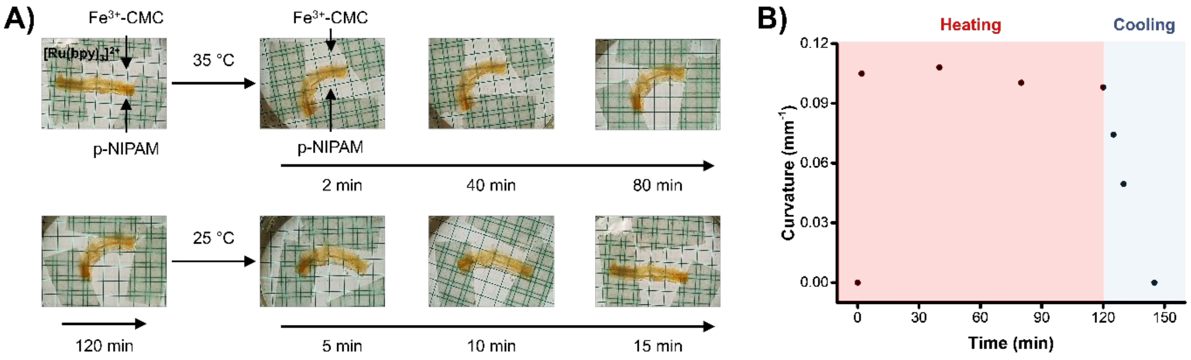

Figure S13. (A) Time dependent mechanical bending of the p-NIPAM/Fe^{3+}-CMC bilayer device upon thermal triggering of the device to 35 °C, followed by cooling to 25 °C and recovery of the bilayer linear device. (B) Curvature of the p-NIPAM/Fe^{3+}-CMC device upon heating and cooling the device.

After 2 minutes upon heating, the bilayer device undergoes a transition from a linear configuration to a bent configuration, exhibiting a curvature[3] (*1/r*) of ca. 0.1 mm^{-1}. As the solution is maintained at 35 °C, up to two hours, the bilayer device maintains its bent configuration, with a curvature (*1/r*) of ca. 0.1 mm^{-1}. Upon cooling the solution to 25 °C, the bent bilayer device progressively displays a less curved configuration and in 15 minutes, restores its parent linear configuration. The curvatures of the device upon heating and cooling are reported in Figure S13B.

The linear p-NIPAM/Fe^{3+}-CMC bilayer device was immersed in a solution comprising 5 µM $[Ru(bpy)_3]^{2+}$ and 20 mM MES buffer, pH 6.00, degassed by nitrogen, and subjected to an increase in temperature up to 35 °C. After 2 minutes upon heating, the bilayer device undergoes a transition from a linear configuration to a bent configuration, exhibiting a curvature (*1/r*) of ca. 0.1 mm^{-1}. The device was illuminated for 20 minutes under N_2 atmosphere, providing a highly curled structure, as depicted in Figure S14A.

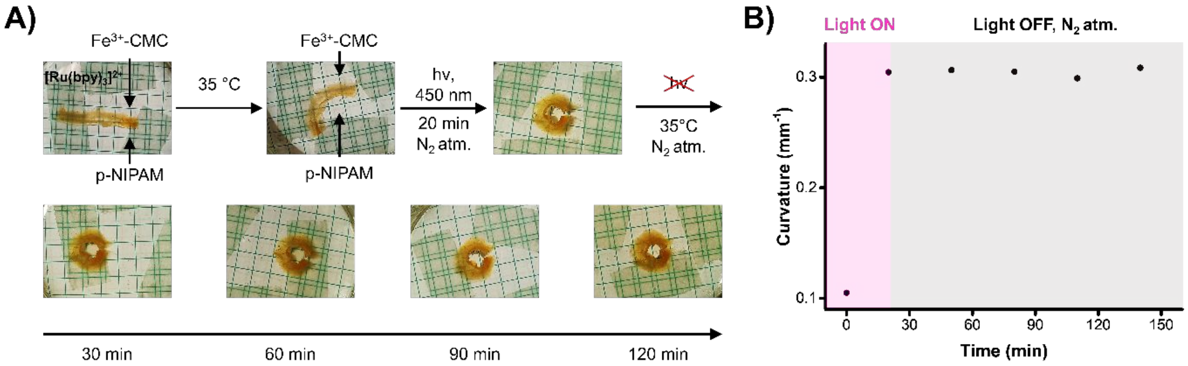

Figure S14. (A) Time dependent mechanical bending of the p-NIPAM/Fe^{3+}-CMC bilayer device upon thermal triggering of the device to 35 °C, followed by 20 min LED irradiation (λ = 450 nm, 20 mW·cm^{-2}) under nitrogen atmosphere. (B) Curvature of the p-NIPAM/Fe^{3+}-CMC device upon heating and illumination of the device under inert atmosphere.

After switching off the light, the device is kept at 35 °C for 2 hours under N_2 atmosphere, maintaining its highly curved structure, with a curvature (*1/r*) of ca. 0.30 mm^{-1}. The curvatures of the device kept under N_2 atmosphere is monitored for two hours at 35 °C and reported in Figure S14B.

8. Comments on the Time-Scales Corresponding to the Dissipative Recovery of the Different Gel Matrices Under Aerobic Conditions

The differences in the time-scales observed during the photochemical driven, transient, dissipative decreases in stiffness of the gels, and the concomitant aerobic re-oxidation of the gel frameworks, depend on the experimental conditions employed. For the evaluation of the rheological properties, indeed, the Fe^{3+}-CMC gels are stored in 2 ml solution comprising 5 μM $[Ru(bpy)_3]^{2+}$ and 10 mM MES buffer solution, and the surface exposed to aerobic atmosphere is 1.54 cm^2. The recovery to the parent gel after irradiation, takes ca. four hours to eight hours. For the transient, dissipative load-release, on the other hand, the Fe^{3+}-CMC gels are prepared employing a lower volume of CMC and Fe^{2+} solution and stored in 1 ml solution comprising 5 μM $[Ru(bpy)_3]^{2+}$ and 1 mM MES buffer solution, and the surface exposed to aerobic atmosphere is 0.79 cm^2. The photoinduced reduction of Fe^{3+}-CMC to Fe^{2+}-CMC under these conditions is less effective, due to the lower amount of sacrificial electron donor, therefore, the time for recovery to the parent Fe^{3+}-CMC gel is lower, from ca. 1 hour to 1.5 hours. For the transient, dissipative bending of the bilayer device, the p-NIPAM/Fe^{3+}-CMC gels are prepared employing a lower volume of CMC and Fe^{2+} solution and stored in 10 ml solution comprising 5 μM $[Ru(bpy)_3]^{2+}$ and 20 mM MES buffer solution, and the surface exposed to aerobic atmosphere is 26.41 cm^2. The photoinduced reduction of Fe^{3+}-CMC to Fe^{2+}-CMC under these conditions is more effective, due to the higher amount of sacrificial electron donor, but the higher surface exposed to aerobic atmosphere causes a higher uptake of oxygen, and therefore the time for

recovery to the parent bent curvature at 35 °C of the p-NIPAM/Fe^{3+}-CMC gel is lowered, from ca. 1 hour to 2 hours.

9. References

[1] Desesa, M. A.; Rogers, L. B. Analytical Application of the Absorption Spectra of the Halide Complexes of Heavy Metals : Spectrophotometric Determination of Iron with Hydrochloric Acid. *Anal. Chim. Acta* **1952**, *6*, 534–541.

[2] Kalyanasundaram, K. Photophysics, Photochemistry and Solar Energy Conversion with Tris(Bipyridyl)Ruthenium(II) and its Analogues. *Coord. Chem. Rev.* **1982**, *46*, 159–244.

[3] Davidson-Rozenfeld, G.; Chen, X.; Qin; Y.; Ouyang, Y.; Sohn, Y. S.; Li, Z.; Nechushtai, R.; Willner, I. Stiffness-Switchable, Biocatalytic pH-Responsive DNA-Functionalized Polyacrylamide Cryogels and their Mechanical Applications. *Adv. Funct. Mater.* **2023**, https://doi.org/10.1002/adfm.202306586.
